# The Synthesis of 2-Aminobenzoxazoles Using Reusable Ionic Liquid as a Green Catalyst under Mild Conditions

**DOI:** 10.3390/molecules22040576

**Published:** 2017-04-02

**Authors:** Ya Zhou, Zhiqing Liu, Tingting Yuan, Jianbin Huang, Chenjiang Liu

**Affiliations:** 1 School of Chemistry and Chemical Engineering, Xinjiang University and Key Laboratory of Oil and Gas Fine Chemicals, Ministry of Education, Urumqi 830046, China; zhouya507@126.com (Y.Z.); liuzhiqing507@163.com (Z.L.); Yuantingting507@126.com (T.Y.); huangjianbin507@126.com (J.H.); 2 Physics and Chemistry Detecting Center, Xinjiang University, Urumqi 830046, China

**Keywords:** 2-Aminobenzoxazoles, heterocyclic ionic liquid, recycled

## Abstract

A facile, green, and efficient method for the direct oxidative amination of benzoxazoles using heterocyclic ionic liquid as catalyst has been developed. The reaction proceeded smoothly at room temperature and gave the desirable 2-aminobenzoxazoles with good to excellent yields (up to 97%). The catalyst 1-butylpyridinium iodide can be easily recycled and reused with similar efficacies for at least four cycles.

## 1. Introduction

**Scheme 1:**
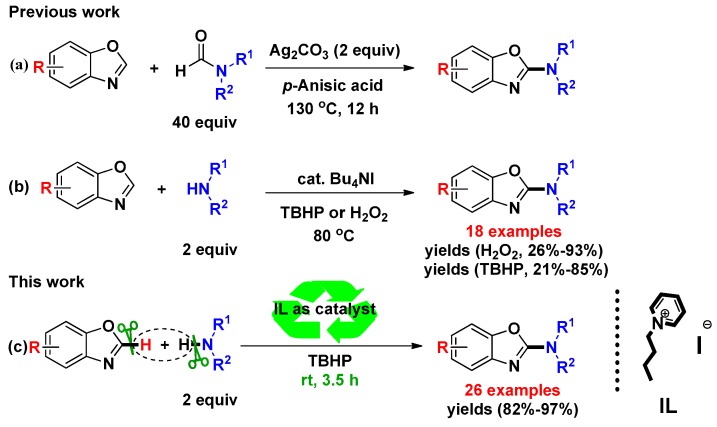
Reported and designed routes for the oxidative amination of benzoxazoles. IL: ionic liquid; TBHP: *tert*-butyl hydroperoxide. Equiv: equivalent.

Amino-substituted azoles and their derivatives are ubiquitous in functional materials, pharmaceuticals, and natural products [1,2,3]. Among them, the 2-aminobenzoxazoles have already been described as potent 5-HT_3_-receptor antagonists [4,5], which are promising targets for the treatment of Alzheimer’s disease and schizophrenia. Furthermore, abundant other drug targets have been addressed by 2-aminobenzoxazoles, such as α7 nicotinic acetylcholine receptor (nAchR) agonist, 5-HT_6_-receptor antagonist, and MK-4305 in clinical trials against insomnia [6,7]. Hence, the development of effective methods to synthesize these compounds has attracted great attention. In this regard, direct C–H amination reaction displaying an advantage in atom efficiency has been pioneered by Cho (Scheme 1a), Monguchi, Wang, Kawano, Miyasaka, Xie and others in the past decade [8,9,10,11,12,13,14,15,16,17,18,19,20]. However, most of these methods could be realized only in the presence of copper, silver, manganese, iron, cobalt. The toxicity and expense of transition metals might limit practical applications.

Recently, metal-free-catalyzed oxidative coupling reactions have undergone rapid advances in order to solve drawbacks of transition metal catalysis [21,22,23,24,25]. Thus, the discovery of green and sustainable oxidative C–H amination of benzoxazoles under metal-free conditions will be of great value. Hypervalent iodine compounds have gained much attention in organic synthesis as versatile and powerful oxidants [26,27,28,29]. For example, Chang and co-workers [30] employed stoichiometric amounts of PhI(OAc)_2_, and Bhanage et al. [31]. used 2-iodoxybenzoic acid (IBX) to get direct oxidative C–H bond amination of benzoxazoles. Moreover, much progress in the direct oxidative amination of benzoxazoles has been achieved through the use of a combination of an iodine source and oxidant [32,33,34,35,36,37,38]. For instance, a metal-free approach for the oxidative amination of benzoxazoles using molecular iodine as a catalyst and *tert*-butyl hydroperoxide (TBHP) as an oxidant was reported by Lamani and Prabhu [32]. At the same time, another report was presented by Nachtsheim’s group on the oxidative C–H amination of benzoxazoles under metal-free conditions using tetrabutylammonium iodide (TBAI) as catalyst and aqueous solutions of H_2_O_2_ or TBHP as co-oxidant at 80 °C (Scheme 1b) [33]. These catalytic systems effectively overcome some drawbacks of using stoichiometric amounts of hypervalent iodine compounds. Nevertheless, these approaches still suffer from minor drawbacks, such as high temperature, long reaction time, and low yields. Therefore, designing a highly mild, efficient, green, and recyclable catalyst system for the oxidative C–H amination of benzoxazoles under metal-free conditions at room temperature is desperately needed.

Ionic liquids (ILs) have attracted continuing interest from the majority of chemists. ILs were usually used as green reaction media because of their unique chemical and physical properties, such as high thermal stabilities, negligible vapor pressures, and nonflammability [39,40,41,42,43,44]. Recently, ILs have been employed as solvents in transition-metal-catalyzed C–H activation reactions [45,46,47,48,49]. However, it is important to note that there are few examples of the C–H bond activation reaction using classical heterocyclic ionic liquids as promoters or catalysts. We recently developed the first oxidative cross-coupling reaction for C–C bond formation promoted by ionic liquid 1,3-dibutyl-1*H*-benzo[*d*][1,2,3]triazol-3-ium bromide [50]. Soon after, the first study using IL-catalyzed C–H activation reaction for C–N bond formation has been disclosed [51]. Inspired by these works, we herein report a mild, efficient, and metal-free strategy for C–H oxidative amination of benzoxazoles by using heterocyclic ionic liquid 1-butylpyridinium iodide ([BPy]I) as catalyst, TBHP as oxidant, and acetic acid as an additive at room temperature (Scheme 1c).

## 2. Results and Discussion

The investigation started with the reaction of benzoxazole (**1a**) and morpholine (**2a**) as model substrates (Table 1). In the presence of 5 mol% [BPy]I, 1.5 equiv. of TBHP, and 3 equiv. of acetic acid, the reaction of **1a** and **2a** was proceeded at room temperature for 7 h to give 2-morpholinobenzo[*d*]oxazole (**3a**) in 94% yield (Table 1, entry 1). Encouraged by the result, further optimization of the reaction conditions was carried out. Unfortunately, when other ionic liquids (e.g., 1-butylpyridinium chloride ([BPy]Cl) and 1-butylpyridinium bromide ([BPy]Br)) were in this reaction system or the system was run in the absence of catalyst, the response almost did not give satisfactory results (Table 1, entries 2–4). Considering that ionic liquids have the advantage of being reusable, the dosage of the [BPy]I was studied in an effort to reduce the reaction time. Gratifyingly, when the amount of the [BPy]I was increased from 5 mol% to 15 mol% (Table 1, entries 1, 6), the reaction time decreased from 7 h to 3.5 h. However, when the dosage of the [BPy]I was increased to 20 mol% (Table 1, entry 7), the reaction time did not reduce. Thus, 15 mol% of [BPy]I was an adequate choice of catalyst for the reaction to achieve high yields with less reaction time. Furthermore, four kinds of organic solvents were investigated, and acetonitrile was found to be the optimal solvent (Table 1, entries 6, 8–10). In water and under solvent-free condition, product **3a** was obtained in lower yields of 57% and 51%, respectively (Table 1, entries 11–12). Other oxidants, such as benzoyl peroxide (BPO), m-chloroperoxybenzoic acid (*m*-CPBA), di-*tert*-butyl peroxide (DTBP), and H_2_O_2_ were used instead of TBHP (Table 1, entries 13–16), the effect of reactions decreased dramatically and TBHP was proven to be the best oxidant.

**Scheme 2:**
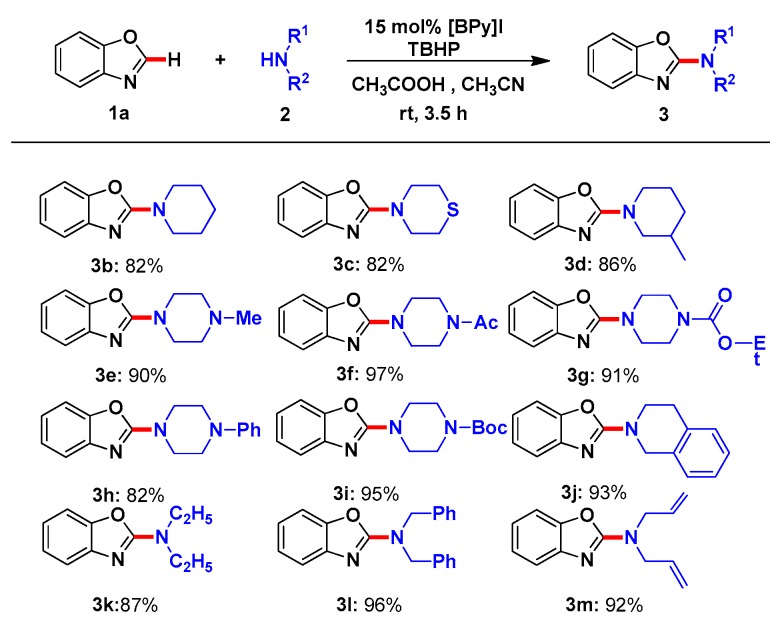
Amination reaction of benzoxazole with various secondary amines. Reaction conditions: **1** (0.672 mmol), **2** (1.344 mmol), oxidant (1.008 mmol), acetic acid (2.016 mmol), [BPy]I (15 mol%), CH_3_CN (2 mL), at room temperature, 3.5 h. Isolated yield. [BPy]I: 1-butylpyridinium iodide.

Under optimized reaction conditions, benzoxazole (**1a**) and various cyclic or acyclic secondary amines (**2**) were investigated to examine the scope of the process. The results are listed in Scheme 2. Piperidine, thiomorpholine, 3-methylpiperidine, and 1-methylpiperazine reacted smoothly with benzoxazole to form the corresponding amination products **3b**, **3c**, **3d,** and **3e** in good to excellent yields (Scheme 2). The introduction of heteroatom and substituent on piperidine had no impact on the reaction system. Subsequently, it was found that both electron-donating and electron-withdrawing groups on the piperazine—such as methyl, phenyl, acetyl, ethoxycarbonyl, and *tert*-butoxycarbonyl—reacted effectively with benzoxazole (**1a**) to provide the respective aminobenzoxazoles (Scheme 2, **3e**: 90%, **3f**: 97%, **3g**: 91%, **3h**: 82%, **3i**: 95%). As is well known, 2-(*N*-alkylpiperazyl)benzoxazoles of this type were already described as potent 5-HT_3_-receptor antagonists [2]. The coupling of benzoxazole proceeded successfully with 1,2,3,4-tetrahydroisoquinoline to give the desired aminated product **3j** in 93% yield (Scheme 2). In addition to cyclic amines, acyclic secondary amines such as diethylamine, dibenzylamine, and diallylamine reacted well with benzoxazole to produce the desired products **3k**–**3m** in good to excellent yields (Scheme 2). To our delight, oxidations or halogenations of the methylene units, the aromatic ring, or the double bonds did not occur. It should be mentioned that *N*,*N*-diallylbenzoxazol-2-amine (**3m**) was regarded as one of the useful organic synthetic intermediates.

**Scheme 3:**
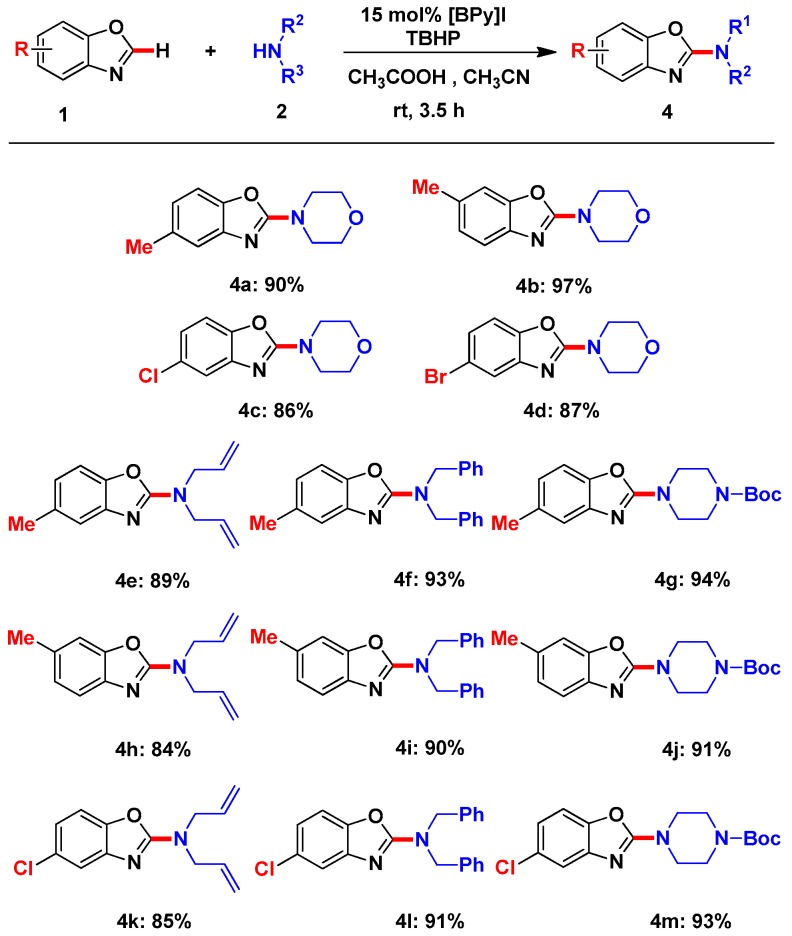
Amination reaction of various benzoxazoles with secondary amines. *^a^* Reaction conditions: (**1**) (0.672 mmol); (**2**) (1.344 mmol), oxidant (1.008 mmol), acetic acid (2.016 mmol), [BPy]I (15 mol%), CH_3_CN (2 mL), at room temperature, 3.5 h. Isolated yield.

To further study the potential of our method, we turned our attention to the amination reaction between various benzoxazoles and morpholine. The results are summarized in Scheme 3. Methyl- and halogen-substituted benzoxazoles could be easily aminated to generate the desired products **4a**–**4d** in up to 97% yield with perfect regioselectivity. To expand the potential of the present synthetic methodology, a diversity of benzoxazoles including 5-methylbenzoxazole, 6-methylbenzoxazole, and 5-chlorobenzoxazole reacted satisfactorily with various linear and cyclic secondary amines such as diallylamine, dibenzylamine, and *tert*-butyl piperazine-1-carboxylate to furnish the corresponding 2-aminobenzothiazole derivatives **4e**–**4i** in good to excellent yields (84%–94%).

**Scheme 4:**
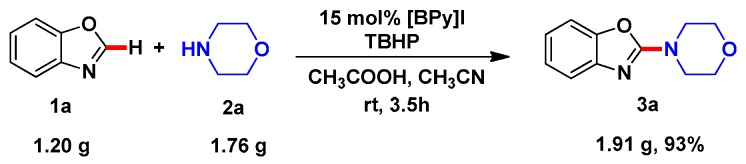
Gram-scale oxidative amination of benzoxazoles and morpholine.

To demonstrate the potential industrial utility of our protocol, the [BPy]I-catalyzed scale-up reaction was performed. The direct oxidative amination of benzoxazole (**1a**) with morpholine was easily carried out under the standard reaction conditions to generate the desired product **3a** in 93% isolated yield (Scheme 4).

**Figure 1:**
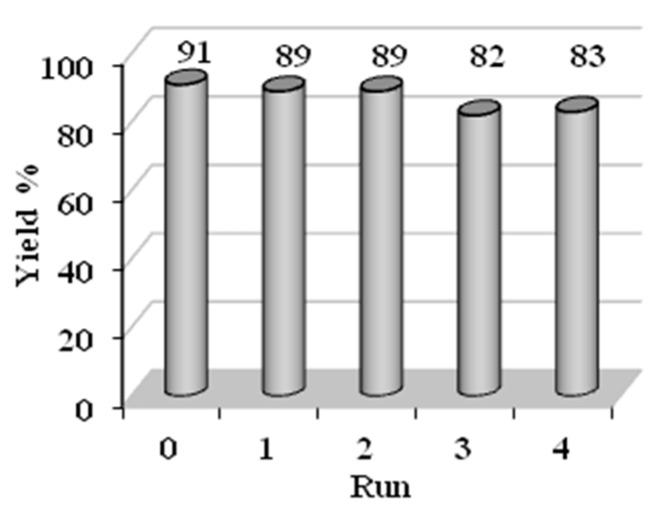
Recycling reactions.

Ionic liquids have several advantages compared with other catalysts; for example, they are recyclable and environmentally friendly. The coupling reaction of 5-methylbenzoxazole and morpholine was chosen as a model system to study the reusability of the [BPy]I as catalyst under the standard reaction conditions. After the reaction was finished, ethyl acetate and water were added, and then the [BPy]I was recovered from the water layer and reused after drying in vacuo. The [BPy]I was utilized repeatedly at least four times without any significant loss of activity (Figure 1).

In order to elucidate the reaction mechanism, control experiments were carried out. When the radical scavenger BHT (2,6-di-*tert*-butyl-4-methylphenol, 3 equiv.), ethene-1,1-diyldibenzene (3 equiv.), or TEMPO (2,2,6,6-tetramethylpiperidine-*N*-oxyl, 3 equiv.) were added to the reaction mixture under the optimal conditions, the yield of **3a** was not decreased. Thus, the reaction may not be a radical reaction. Subsequently, *N*-iodomorpholine hydroiodide was prepared. Then, the reaction of benzoxazole (0.672 mmol, 1 equiv.) and *N*-iodomorpholine hydroiodide (0.672 mmol, 1 equiv.) with acetic acid (2.016 mmol, 3 equiv.) as an additive in 2 mL CH_3_CN at room temperature for 3.5 h afforded the desired product **3a** in 25% yield (Table 2, entry 1). When the dosage of *N*-iodomorpholine hydroiodide was increased from 1 equiv. to 2 equiv., the desired amination product was obtained in 53% yield (Table 2, entry 2). It is worth noting that the reaction of **1a** and **2a** underwent smoothly in the presence of 15 mol% *N*-iodomorpholine hydroiodide, 1.5 equiv. TBHP, and 3 equiv. acetic acid to give 2-morpholinobenzo[*d*]oxazole (**3a**) in 95% yield (Table 2, entry 3). All of the above results indicate that the activation of the morpholine (**2a**) via in situ preparation of a highly reactive N-I bond from [BPy]I and TBHP seems plausible.

**Scheme 5:**
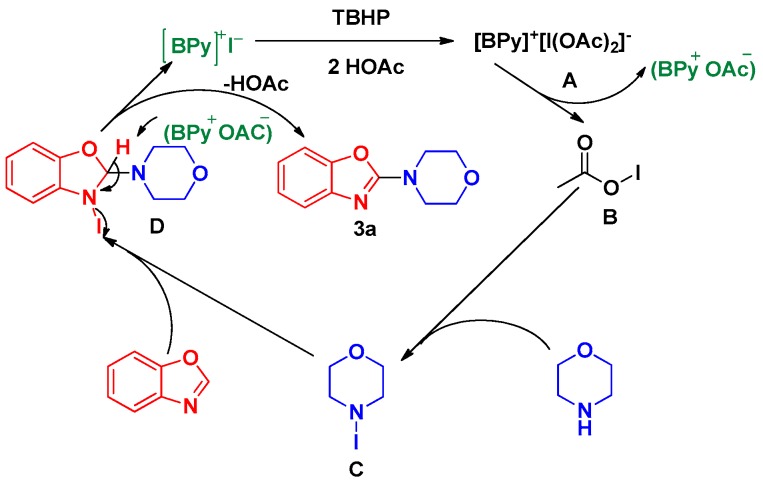
The proposed mechanism. HOAc: acetic acid.

Based on the above results and existing literature [33], a plausible mechanism is proposed as shown in Scheme 5. Initially, [BPy]I is oxidized by TBHP to form [BPy]^+^[I(OAc)_2_]^−^ (**A**) in the presence of acetic acid, and then releases acetylhypoiodite **B**. Subsequently, **C** is generated by combining morpholine with highly potent I^+^ source **B**. Immediately following, the reaction of C and benzoxazole affords **D**. Finally, the desired amination reaction product **3a** can be obtained after the elimination of hydrogen iodide.

## 3. Experimental Section

A reaction vessel was charged with acetic acid (2.016 mmol) and TBHP (70% in water, 1.008 mmol) in acetonitrile (2 mL). After the addition of [BPy]I (0.1008 mmol), benzoxazole (0.672 mmol) and secondary amines (1.344 mmol) were added. Then, the reaction mixture was stirred at room temperature for 3.5 h. After the reaction finished, the mixture was extracted with dichloromethane (5 × 10 mL), and the combined organic phases were dried over anhydrous Na_2_SO_4_. The solvent was evaporated under vacuum, and the crude residue was purified by column chromatography on silica gel. Aqueous phase was dried in a vacuum evaporator to recover the ionic liquid and directly reused in subsequent runs.

## 4. Conclusions

In summary, we have found an IL-catalyzed direct oxidative amination of benzoxazoles under metal-free conditions at room temperature. This mild catalytic system is suitable for the oxidative amination reactions between a wide range of secondary amines and benzoxazoles. In addition, the inexpensive and environmentally friendly ionic liquid [BPy]I can be easily recycled and reused for four runs without any obvious loss of catalytic activity. ILs-catalyzed C–H bond activation is ongoing in our group.

## Figures and Tables

**Table 1:**
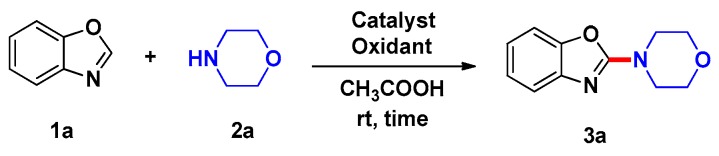
Optimizing the reaction conditions. *^a^*

Entry	Catalyst (mol%)	Oxidant *^b^*	Solvent	Time (h)	Yield (%) *^c^*
**1**	[BPy]I (5)	TBHP	CH_3_CN	7	94
**2**	[BPy]Cl (5)	TBHP	CH_3_CN	7	Trace
**3**	[BPy]Br (5)	TBHP	CH_3_CN	7	N.R. *^d^*
**4**	-	TBHP	CH_3_CN	7	N.R.
**5**	[BPy]I (10)	TBHP	CH_3_CN	5	91
**6**	**[BPy]I (15)**	**TBHP**	**CH_3_CN**	**3.5**	**94**
**7**	[BPy]I (20)	TBHP	CH_3_CN	3.5	94
**8 ^c^**	[BPy]I (15)	TBHP	CH_2_Cl_2_	3.5	88
**9**	[BPy]I (15)	TBHP	THF	3.5	85
**10**	[BPy]I (15)	TBHP	toluene	3.5	86
**11**	[BPy]I (15)	TBHP	H_2_O	3.5	57
**12**	[BPy]I (15)	TBHP	Neat	3.5	51
**13**	[BPy]I (15)	BPO	CH_3_CN	3.5	65
**14**	[BPy]I (15)	*m*-CPBA	CH_3_CN	3.5	N.R.
**15**	[BPy]I (15)	DTBP	CH_3_CN	3.5	Trace
**16**	[BPy]I (15)	H_2_O_2_	CH_3_CN	3.5	79

*^a^* Reaction conditions: **1a** (0.672 mmol), **2a** (1.344 mmol), oxidant (1.008 mmol), acetic acid (2.016 mmol) in 2 mL solvent at room temperature. *^b^* TBHP: *tert*-butyl hydroperoxide 70% in water, BPO: benzoyl peroxide, *m*-CPBA: m-chloroperoxybenzoic acid, DTBP: di-*tert*-butyl peroxide, H_2_O_2_ 30% in water, THF: tetrahydrofuran. *^c^* Isolated yield. *^d^* Not reaction. The optimized reaction conditions were 15 mol% [BPy]I as catalyst, 1.5 equiv. TBHP as oxidant, and 3 equiv. acetic acid as additive in 2 mL CH_3_CN at room temperature for 3.5 h.

**Table 2:**
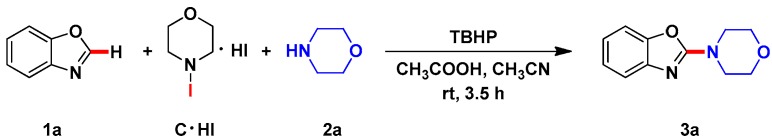
Reactivity of *N*-Iodomorpholine Hydroiodide **C^.^HI** *^a^*.

Entry	1a (Equiv.)	C^.^HI (Equiv.)	2a (Equiv.)	TBHP (Equiv.)	Yield (%) *^b^*
1	1	1	0	0	25
2	1	2	0	0	53
3	1	0.15	2	1.5	95

*^a^* Reaction conditions: **1a** (0.672 mmol), acetic acid (2.016 mmol), CH_3_CN (2 mL), room temperature, 3.5 h; *^b^* Isolated yield.

## Supplementary Files

Supplementary File 1
