# Supplementary material for: The Synthesis of 2-Aminobenzoxazoles Using Reusable Ionic Liquid as a Green Catalyst under Mild Conditions
Source: Molecules. 2017 Apr 2;22(4):576. doi: 10.3390/molecules22040576 (PMC6154564; doi:10.3390/molecules22040576)

Supporting Information

The Synthesis of 2-Aminobenzoxazoles Using Reusable Ionic Liquid as an Green Catalyst under Mild Conditions

Ya Zhou†, Zhiqing Liu†, Tingting Yuan, Jianbin Huang

and Chenjiang Liu,*

† The two authors contributed equally to this paper.

The Key Laboratory of Oil and Gas Fine Chemicals, Ministry of Education & Xinjiang Uygur Autonomous Region, Urumqi Key Laboratory of Green Catalysis and Synthesis Technology, School of Chemistry and Chemical Engineering, Physics and Chemistry Detecting Center, Xinjiang University, Urumqi 830046, P. R. of China.

pxylcj@126.com

Contents

1. General methods S2

2. General experimental procedure S2

3. Characterization data of 2-aminobenzoxazoles (**3a-3m**, **4a-4m**) S3

4. References...S9

5. ^{1}H and ^{13}C NMR spectra of the compounds S10

Ⅰ. General methods

All reagents and solvents were purchased from commercial sources (Adamas-beta, TCI, Alfa Aesar and Ark) and used without further purification. Analytical thin layer chromatography (TLC) was performed on 0.25 mm silica gel 60 F_{254} plates. Visualization on TLC was achieved by the use of UV light (254 nm). Column chromatography was undertaken on silica gel (300-400 mesh) using a proper eluent. ^{1}H and ^{13}C NMR spectra were collected on 400 MHz NMR spectrometers (Varian Inova-400). Chemical shifts for protons were reported in parts per million (ppm) downfield from tetramethylsilane and were referenced to residual protium in the NMR solvents ($CDCl_3$ = δ 7.26). Chemical shifts for carbon resonances were reported in parts per million (ppm) downfield from tetramethylsilane and were referenced to the carbon resonances of the solvents ($CDCl_3$ = δ 77.00). The following abbreviations were used to describe peak splitting patterns when appropriate: br = broad, s = singlet, d = doublet, t = triplet, q = quartet, dt = doublet of triplet, m = multiplet. Coupling constants *J* were reported in hertz unit (Hz). High-resolution mass spectra (HRMS) were recorded on Thermo Q-Exactive.

II. General experimental procedure

A reaction vessel was charged with acetic acid (2.016 mmol) and TBHP (70% in water, 1.008 mmol) in acetonitrile (2 mL). After the addition of [Bpy]I (0.1008 mmol), benzoxazole (0.672 mmol) and secondary amines (1.344 mmol) were added. Then the reaction mixture was stirred at room temperature for 3.5 hours. After the reaction finished, the mixture was extracted with dichloromethane (5 × 10 mL), and the combined organic phases were dried over anhydrous Na_2SO_4. The solvent was evaporated under vacuo, and the crude residue was purified by column chromatography on silica gel. Aqueous phase was dried in a vacuum evaporator to recover the ionic liquid and directly reused in subsequent runs.

III Characterization data of 2-aminobenzoxazoles (3a-3m, 4a-4m)

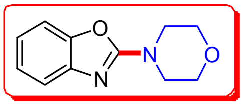

2-Morpholinobenzo[*d*]oxazole **3a**[1]

White solid (yield: 94%), eluent for column chromatography hexane : ethyl acetate v/v 4 : 1; ^{1}H NMR (400 MHz, $CDCl_3$): δ 7.39-7.36 (m, 1H), 7.28-7.25 (m, 1H), 7.18 (dt, *J*=7.7 Hz, 1.1 Hz, 1H), 7.04 (dt, *J*=7.8 Hz, 1.2 Hz, 1H), 3.82-3.80 (m, 4H), 3.69-3.67 (m, 4H); ^{13}C NMR (100 MHz, $CDCl_3$): δ 162.1, 148.7, 142.8, 124.1, 120.9, 116.5, 108.8, 66.2, 45.7.

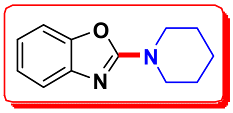

2-(Piperidin-1-yl)benzo[*d*]oxazole **3b**[1]

Faint yellow solid (yield: 82%), eluent for column chromatography hexane : ethyl acetate v/v 10 : 1; ^{1}H NMR (400 MHz, $CDCl_3$): δ 7.35-7.33 (m, 1H), 7.23-7.21 (m, 1H), 7.14 (dt, *J*= 7.7 Hz, 1.1 Hz, 1H), 6.99 (dt, *J*= 7.8 Hz, 1.2 Hz, 1H), 3.64-3.63 (m, 4H), 1.66 (s, 6H); ^{13}C NMR (100 MHz, $CDCl_3$): δ 162.4, 148.7, 143.4, 123.8, 120.2, 116.0, 108.5, 46.6, 25.2, 24.1.

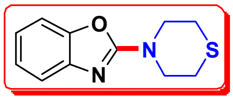

2-Thiomorpholinobenzo[*d*]oxazole **3c**[2]

Faint yellow solid (yield: 82%), eluent for column chromatography hexane : ethyl acetate v/v 10 : 1; ^{1}H NMR (400 MHz, $CDCl_3$): δ 7.40-7.38 (m, 1H), 7.28-7.25 (m, 1H), 7.17 (dt, *J*= 8.0 Hz, 1.1 Hz, 1H), 7.03 (dt, *J*= 8.0 Hz, 1.2 Hz, 1H), 4.04-4.01 (m, 4H), 2.76-2.73 (m, 4H); ^{13}C NMR (100 MHz, $CDCl_3$): δ 161.5, 148.6, 142.6, 124.1, 120.9, 116.3, 108.8, 48.1, 26.7.

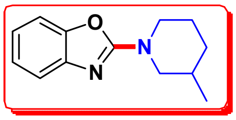

2-(3-Methylpiperidin-1-yl)benzo[*d*]oxazole **3d**[3]

White solid (yield: 86%), eluent for column chromatography hexane : ethyl acetate v/v 8 : 1; ^{1}H NMR (400 MHz, $CDCl_3$): δ 7.36-7.34 (m, 1H), 7.24-7.22 (m, 1H), 7.14 (dt, *J*= 7.7 Hz, 1.1 Hz, 1H), 7.00 (dt, *J*= 7.8 Hz, 1.2 Hz, 1H), 4.22-4.15 (m, 2H), 3.06-2.99 (m, 1H), 2.73-2.67 (m, 1H), 1.88-1.56 (m, 4H), 1.20-1.10 (m, 1H), 0.97-0.95 (d, 3H); ^{13}C NMR (100 MHz, $CDCl_3$): δ 162.1, 148.5, 143.0, 123.9, 120.4, 115.9, 108.6, 53.0, 46.1, 32.6, 30.6, 24.7, 18.9.

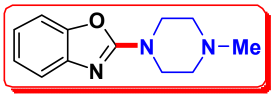

2-(4-Methylpiperazin-1-yl)benzo[*d*]oxazole **3e**[1]

Faint yellow solid (yield: 90%), eluent for column

chromatography dichloromethane : methanol v/v 15 : 1; ^{1}H NMR (400 MHz, $CDCl_3$): δ 7.34-7.32 (m, 1H), 7.23-7.21 (m, 1H), 7.13 (dt, J= 7.7 Hz, 1.1 Hz, 1H), 6.99 (dt, J= 7.8 Hz, 1.2 Hz, 1H), 3.69 (t, J = 5.2 Hz, 4H), 2.49 (t, J = 5.2 Hz 4H), 2.31 (s, 3H); ^{13}C NMR (100 MHz, $CDCl_3$): δ 162.2, 148.7, 143.1, 124.0, 120.6, 116.3, 108.7, 54.2, 46.2, 45.5.

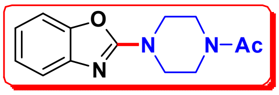

1-(4-(Benzo[*d*]oxazol-2-yl)piperazin-1-yl)ethan-1-one **3f**[1]

Faint yellow solid (yield: 97%), eluent for column chromatography hexane : ethyl acetate v/v 1 : 3; ^{1}H NMR (400 MHz, $CDCl_3$): δ 7.39-7.37 (m, 1H), 7.29-7.27 (m, 1H), 7.19 (dt, J = 7.7 Hz, 1.1 Hz, 1H), 7.06 (dt, J = 7.7 Hz, 1.2 Hz, 1H), 3.78-3.68 (m, 6H), 3.62-3.60 (m, 2H), 2.16 (s, 3H); ^{13}C NMR (100 MHz, $CDCl_3$): δ 169.2, 161.7, 148.7, 142.7, 124.2, 121.1, 116.6, 109.0, 45.7, 45.6, 45.4, 40.8, 21.4.

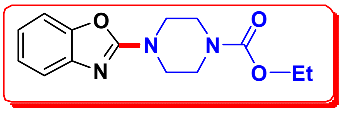

Ethyl 4-(benzo[*d*]oxazol-2-yl)piperazine-1-carboxylate **3g**[4]

Yellow solid (yield: 91%), eluent for column chromatography hexane : ethyl acetate v/v 6 : 1; ^{1}H NMR (400 MHz, $CDCl_3$): δ 7.38-7.36 (m, 1H), 7.26-7.25 (m, 1H), 7.18 (dt, J= 7.7 Hz, 1.1 Hz, 1H), 7.04 (dt, J= 7.8 Hz, 1.2 Hz, 1H), 4.19 (q, J= 7.1 Hz, 2H), 3.70-3.61 (m, 8H), 1.29 (t, J= 7.1 Hz 3H); ^{13}C NMR (100 MHz, $CDCl_3$): δ 161.8, 155.3, 148.7, 142.7, 124.0, 120.9, 116.4, 108.8, 61.7, 45.3, 43.0, 14.6.

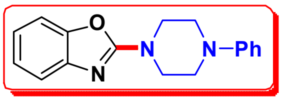

2-(4-Phenylpiperazin-1-yl)benzo[*d*]oxazole **3h**[5]

Yellow solid (yield: 82%), eluent for column chromatography hexane : ethyl acetate v/v 4 : 1; ^{1}H NMR (400 MHz, $CDCl_3$): δ 7.39-7.37 (m, 1H), 7.32-7.25 (m, 3H), 7.18 (dt, J= 7.7 Hz, 1.1 Hz, 1H), 7.04 (dt, J= 7.8 Hz, 1.2 Hz, 1H), 7.00-6.96 (m, 2H), 6.94-6.91 (m, 1H), 3.87-3.85 (m, 4H), 3.31-3.29 (m, 4H); ^{13}C NMR (100 MHz, $CDCl_3$): δ 162.1, 151.1, 148.8, 143.0, 129.3, 124.1, 120.8, 120.7, 116.9, 116.4, 108.8, 49.2, 45.6.

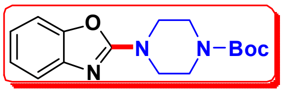

Tert-butyl 4-(benzo[*d*]oxazol-2-yl)piperazine-1-carboxylate **3i**[5]

White solid (yield: 95%), eluent for column chromatography hexane : ethyl acetate v/v 10 : 1; ^{1}H NMR (400 MHz, $CDCl_3$): δ 7.40-7.38 (m, 1H), 7.28-7.26 (m, 1H), 7.19 (dt, J= 7.7 Hz, 1.1 Hz, 1H), 7.06 (dt, J= 7.8 Hz, 1.1 Hz, 1H),

3.71-3.69 (m, 4H), 3.59-3.56 (m, 4H), 1.49 (s, 9H); ^{13}C NMR (100 MHz, $CDCl_3$): δ 161.9, 154.5, 148.7, 142.8, 124.1, 120.9, 116.4, 108.8, 80.4, 45.4, 28.4.

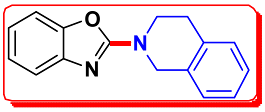

2-(3,4-Dihydroisoquinolin-2(1*H*)-yl)benzo[*d*]oxazole **3j**[4]

Yellow solid (yield: 93%), eluent for column chromatography hexane : ethyl acetate v/v 6 : 1; ^{1}H NMR (400 MHz, $CDCl_3$): δ 7.39-7.37 (m, 1H), 7.28-7.26 (m, 1H), 7.24-7.14 (m, 5H), 7.01 (dt, *J*= 7.8 Hz, 1.2 Hz, 1H), 4.84 (s, 2H), 3.93 (t, *J* = 6.0 Hz, 2H), 2.98 (t, *J* = 5.9 Hz, 2H); ^{13}C NMR (100 MHz, $CDCl_3$): δ 162.0, 148.8, 143.2, 134.0, 132.3, 128.8, 126.8, 126.5, 126.3, 124.0, 120.5, 116.2, 108.7, 47.1, 43.1, 28.4.

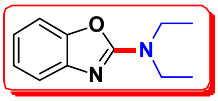

N,N-Diethylbenzo[*d*]oxazol-2-amine **3k**[1]

Light yellow liquid (yield: 87%), eluent for column chromatography hexane : ethyl acetate v/v 10 : 1; ^{1}H NMR (400 MHz, $CDCl_3$): δ 7.36-7.34 (m, 1H), 7.24-7.22 (m, 1H), 7.13 (dt, *J* = 7.7 Hz, 1.1 Hz, 1H), 6.97 (dt, *J* = 7.7 Hz, 1.2 Hz, 1H), 3.57 (q, *J* = 7.1 Hz, 4H), 1.26 (t, *J* = 7.1 Hz, 6H); ^{13}C NMR (100 MHz, $CDCl_3$): δ 162.2, 148.8, 143.6, 123.8, 119.9, 115.8, 108.5, 42.9, 13.5.

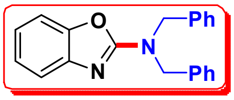

N,N-Dibenzylbenzo[*d*]oxazol-2-amine **3l**[1]

White solid (yield: 96%), eluent for column chromatography hexane : ethyl acetate v/v 10 : 1; ^{1}H NMR (400 MHz, $CDCl_3$): δ 7.42-7.40 (m, 1H), 7.34-7.24 (m, 11H), 7.19 (dt, *J* = 7.7 Hz, 1.1 Hz, 1H), 7.07-7.02 (m, 1H), 4.71 (s, 4H); ^{13}C NMR (100 MHz, $CDCl_3$): δ 163.2, 149.0, 143.5, 136.3, 128.7, 127.9, 127.7, 124.1, 120.5, 116.3, 108.8, 50.3.

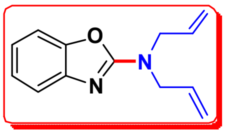

N,N-Diallylbenzo[*d*]oxazol-2-amine **3m**[1]

Light yellow liquid (yield: 92%), eluent for column chromatography hexane : ethyl acetate v/v 10 : 1; ^{1}H NMR (400 MHz, $CDCl_3$): δ 7.38-7.35 (m, 1H), 7.26-7.24 (m, 1H), 7.15 (dt, *J* = 7.7 Hz, 1.1 Hz, 1H), 7.02-7.00 (m, 1H), 5.91-5.83 (m, 2H), 5.27-5.22 (m, 4H), 4.15 (dt, *J* = 5.7 Hz, 1.3 Hz, 4H); ^{13}C NMR (100 MHz, $CDCl_3$): δ 162.3, 148.9, 143.4, 132.6, 123.9, 120.4, 117.9, 116.2, 108.7, 49.3.

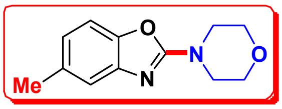

5-Methyl-2-morpholinobenzo[*d*]oxazole **4a**[1]

White solid (yield: 90%), eluent for column chromatography hexane : ethyl acetate v/v 4 : 1; ^{1}H NMR (400 MHz, $CDCl_3$): δ 7.17 (s, 1H), 7.13 (d, *J* = 8.1 Hz, 1H), 6.86-6.83(m, 1H), 3.83-3.80 (m, 4H), 3.69-3.64 (m, 4H), 2.40 (s, 3H); ^{13}C NMR (100 MHz, $CDCl_3$): δ 162.3, 146.9, 142.9, 133.8, 121.6, 116.9, 108.2, 66.2, 45.7, 21.5.

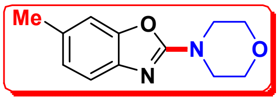

6-Methyl-2-morpholinobenzo[*d*]oxazole **4b**[1]

White solid (yield: 97%), eluent for column chromatography hexane : ethyl acetate v/v 4 : 1; ^{1}H NMR (400 MHz, $CDCl_3$): δ 7.26-7.24 (m, 1H), 7.08 (d, *J* = 0.6 Hz, 1H), 6.99 (dd, *J* = 7.9 Hz, 0.6 Hz, 1H), 3.83-3.80 (m, 4H), 3.68-3.65 (m, 4H), 2.40 (s, 3H); ^{13}C NMR (100 MHz, $CDCl_3$): δ 161.9, 148.9, 140.3, 131.0, 124.8, 115.9, 109.4, 66.2, 45.8, 21.5.

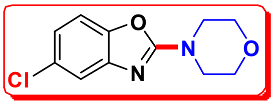

5-Chloro-2-morpholinobenzo[*d*]oxazole **4c**[1]

Faint yellow solid (yield: 86%), eluent for column chromatography hexane : ethyl acetate v/v 10 : 1; ^{1}H NMR (400 MHz, $CDCl_3$): δ 7.32 (d, *J* = 1.9 Hz, 1H), 7.15 (d, *J* = 8.5 Hz, 1H), 6.99 (dd, *J* = 8.5 Hz, 2.1 Hz, 1H), 3.83-3.80 (m, 4H), 3.70-3.67 (m, 4H); ^{13}C NMR (100 MHz, $CDCl_3$): δ 162.8, 147.3, 144.3, 129.4, 120.7, 116.6, 109.3, 66.1, 45.6.

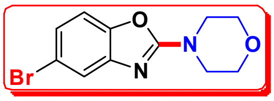

5-Bromo-2-morpholinobenzo[*d*]oxazole **4d**[6]

Yellow solid (yield: 87%), eluent for column chromatography hexane : ethyl acetate v/v 4 : 1; ^{1}H NMR (400 MHz, $CDCl_3$): δ 7.47-7.46 (m, 1H), 7.16-7.10 (m, 2H), 3.83-3.80 (m, 4H), 3.70-3.67 (m, 4H); ^{13}C NMR (100 MHz, $CDCl_3$): δ 162.6, 147.8, 144.7, 123.5, 119.5, 116.8, 109.9, 66.1, 45.6.

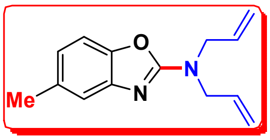

N,N-Diallyl-5-methylbenzo[*d*]oxazol-2-amine **4e**[7]

Light yellow liquid (yield: 89%), eluent for column chromatography hexane : ethyl acetate v/v 6 : 1; ^{1}H NMR (400 MHz, $CDCl_3$): δ 7.18-7.17 (m, 1H), 7.12 (d, *J* = 8.1 Hz, 1H), 6.82-6.80 (m, 1H), 5.92-5.84 (m, 2H), 5.27-5.22 (m, 4H), 4.13 (dt, *J* = 5.7 Hz, 1.4 Hz, 4H), 2.39 (s, 3H); ^{13}C NMR (100 MHz, $CDCl_3$): δ 162.5, 147.1, 143.5, 133.5, 132.7, 121.0, 117.8, 116.6, 108.0, 49.9, 21.5.

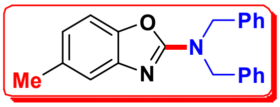

N,N-Dibenzyl-5-methylbenzo[*d*]oxazol-2-amine **4f**[8]

White solid (yield: 93%), eluent for column chromatography hexane : ethyl acetate v/v 6 : 1; ^{1}H NMR (400 MHz, CDCl$_3$): δ 7.40-7.28 (m, 10H), 7.21 (br, 1H), 7.19 (s, 1H), 6.90-6.88 (m, 1H), 4.74 (s, 4H), 2.47 (s, 3H); ^{13}C NMR (100 MHz, CDCl$_3$): δ 163.3, 147.1, 143.6, 136.4, 133.7, 128.7, 127.9, 127.7, 121.2, 116.7, 108.2, 50.3, 21.5.

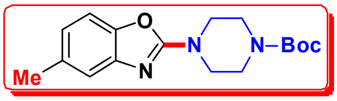

Tert-butyl 4-(5-methylbenzo[*d*]oxazol-2-yl)piperazine-1-carboxylate **4g**[9]

White solid (yield: 94%), eluent for column chromatography hexane : ethyl acetate v/v 4 : 1; ^{1}H NMR (400 MHz, CDCl$_3$): δ 7.19 (s, 1H), 7.14 (d, *J* = 8.1 Hz, 1H), 6.87-6.84(m, 1H), 3.70-3.67 (m, 4H), 3.58-3.55 (m, 4H), 2.39 (s, 3H), 1.49 (s, 9H); ^{13}C NMR (100 MHz, CDCl$_3$): δ 161.7, 154.5, 146.6, 142.0, 134.0, 121.9, 116.7, 108.3, 80.4, 45.6, 28.4, 21.5.

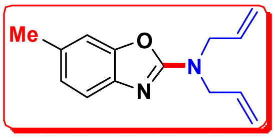

N,N-Diallyl-6-methylbenzo[*d*]oxazol-2-amine **4h**[10]

Light yellow liquid (yield: 84%), eluent for column chromatography hexane : ethyl acetate v/v 6 : 1; ^{1}H NMR (400 MHz, CDCl$_3$): δ 7.24 (d, *J* = 8.0 Hz, 1H), 7.07 (br, 1H), 6.97-6.95 (m, 1H), 5.91-5.84 (m, 2H), 5.25-5.20 (m, 4H), 4.13 (dt, *J* = 5.7 Hz, 1.3 Hz, 4H), 2.39 (s, 3H); ^{13}C NMR (100 MHz, CDCl$_3$): δ 162.1, 149.1, 141.0, 132.7, 130.3, 124.6, 117.8, 115.6, 109.3, 49.9, 21.4.

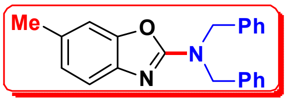

N,N-Dibenzyl-6-methylbenzo[*d*]oxazol-2-amine **4i**

White solid (yield: 90%), eluent for column chromatography hexane : ethyl acetate v/v 6 : 1; ^{1}H NMR (400 MHz, CDCl$_3$): δ 7.34-7.25 (m, 11H), 7.10 (br, 1H), 7.01-6.99 (m, 1H), 4.68 (s, 4H), 2.40 (s, 3H); ^{13}C NMR (100 MHz, CDCl$_3$): δ 162.9, 149.1, 141.0, 136.4, 130.6, 128.7, 127.9, 127.7, 124.7 115.7, 109.4, 50.4, 21.5; $C_{22}H_{20}N_2O$ $[M+H]^+$: 329.16484, found: 329.16443.

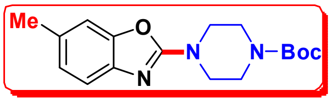

Tert-butyl 4-(6-methylbenzo[*d*]oxazol-2-yl)piperazine-1-carboxylate **4j**[11]

White solid (yield: 91%), eluent for column chromatography hexane : ethyl acetate v/v 4 : 1; ^{1}H NMR (400 MHz, CDCl$_3$): δ 7.30 (d, *J* = 8.0 Hz, 1H), 7.10 (s, 1H), 7.02

(d, J = 8.0 Hz, 1H), 3.73-3.71 (m, 4H), 3.60-3.57 (m, 4H), 2.41 (s, 3H), 1.49 (s, 9H); ^{13}C NMR (100 MHz, $CDCl_3$): δ 161.8, 154.6, 149.0, 140.4, 131.0, 124.8, 115.9, 109.4, 80.4, 45.5, 28.4, 21.5.

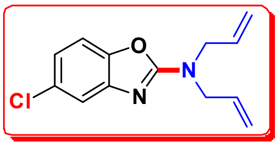

N,N-Diallyl-5-chlorobenzo[*d*]oxazol-2-amine **4k**[7]

Light yellow liquid (yield: 85%), eluent for column chromatography hexane : ethyl acetate v/v 10 : 1; ^{1}H NMR (400 MHz, $CDCl_3$): δ 7.31-7.30 (m, 1H), 7.14-7.12 (m, 1H), 6.97-6.94 (m, 1H), 5.90-5.83 (m, 2H), 5.27-5.22 (m, 4H), 4.14 (dt, J = 5.8 Hz, 1.3 Hz, 4H); ^{13}C NMR (100 MHz, $CDCl_3$): δ 163.1, 147.5, 144.8, 132.3, 129.2, 120.2, 118.1, 116.3, 109.2, 50.0.

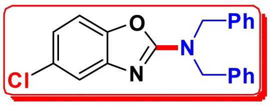

N,N-Dibenzyl-5-chlorobenzo[*d*]oxazol-2-amine **4l**[12]

White solid (yield: 91%), eluent for column chromatography hexane : ethyl acetate v/v 10 : 1; ^{1}H NMR (400 MHz, $CDCl_3$): δ 7.38-7.23 (m, 11H), 7.16 (d, J = 8.5 Hz, 1H), 7.01-6.98 (m, 1H), 4.69 (s, 4H); ^{13}C NMR (100 MHz, $CDCl_3$): δ 163.9, 147.5, 144.9, 136.0, 129.4, 128.8, 127.9, 127.8, 120.3, 116.4, 109.3 50.4.

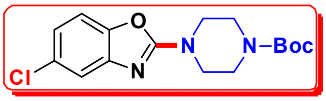

Tert-butyl 4-(5-chlorobenzo[*d*]oxazol-2-yl)piperazine-1-carboxylate **4m**[11]

White solid (yield: 93%), eluent for column chromatography hexane : ethyl acetate v/v 10 : 1; ^{1}H NMR (400 MHz, $CDCl_3$): δ 7.34 (d, J = 1.9 Hz, 1H), 7.16 (d, J = 8.4 Hz, 1H), 7.02-6.99 (m, 1H), 3.71-3.68 (m, 4H), 3.59-3.56 (m, 4H), 1.49 (s, 9H); ^{13}C NMR (100 MHz, $CDCl_3$): δ 162.7, 154.5, 147.3, 144.3, 129.4, 120.7, 116.5, 109.3, 80.5, 45.3, 28.4.

VI. References

1. T. Froehr, C. P. Sindlinger, U. Kloeckner, P. Finkbeiner, B. J. Nachtsheim, *Org. Lett.*, **2011**, *13*, 3754.

2. S. Yotphan, D. Beukeaw, V. Reutrakul, *Synthesis*, **2013**, *45*, 936.

3. X. Wang, D. Q. Xu, C. X. Miao, Q. H. Zhang, W. Sun, *Org. Biomol. Chem.*, **2014**, *12*, 3108.

4. M. Lamani, K. R. Prabhu, *J. Org. Chem.*, **2011**, *76*, 7938.

5. S. Wertz, S. Kodama, A. Studer, *Angew. Chem. Int. Ed.*, **2011**, *50*, 11511.

6. R. Wang, H. Liu, L. Yue, X. K. Zhang, Q. Y. Tan, R. L. Pan, *Tetrahedron Lett.*, **2014**, *55*, 2233.

7. Y. M. Li, J. Liu, Y. S. Xie, R. Zhang, K. Jin, X. N. Wang, C. Y. Duan, *Org. Biomol. Chem.*, **2012**, *10*, 3715.

8. J. Joseph, J. Y. Kim, S. Chang, *Chem. Eur. J.*, **2011**, *17*, 8294.

9. J. Y. Kim, S. H. Cho, J. Joseph, S. Chang, *Angew. Chem. Int. Ed.*, **2010**, *122*, 10095.

10. Y. S. Wagh, D. N. Sawant, B. M. Bhanage, *Tetrahedron Lett.*, **2012**, *53*, 3482.

11. C. L. Cioffi, J. J. Lansing, H.Yüksel, *J. Org. Chem.*, **2010**, *75*, 7942.

12. Y. Masatoshi, T. K. H. Yasuo, H. Kuniko, *Chem. Pharm. Bull.*, **1983**, *32*, 3053.

V. ^{1}H and ^{13}C NMR spectra of the compounds

2-Morpholinobenzo[*d*]oxazole (3a)

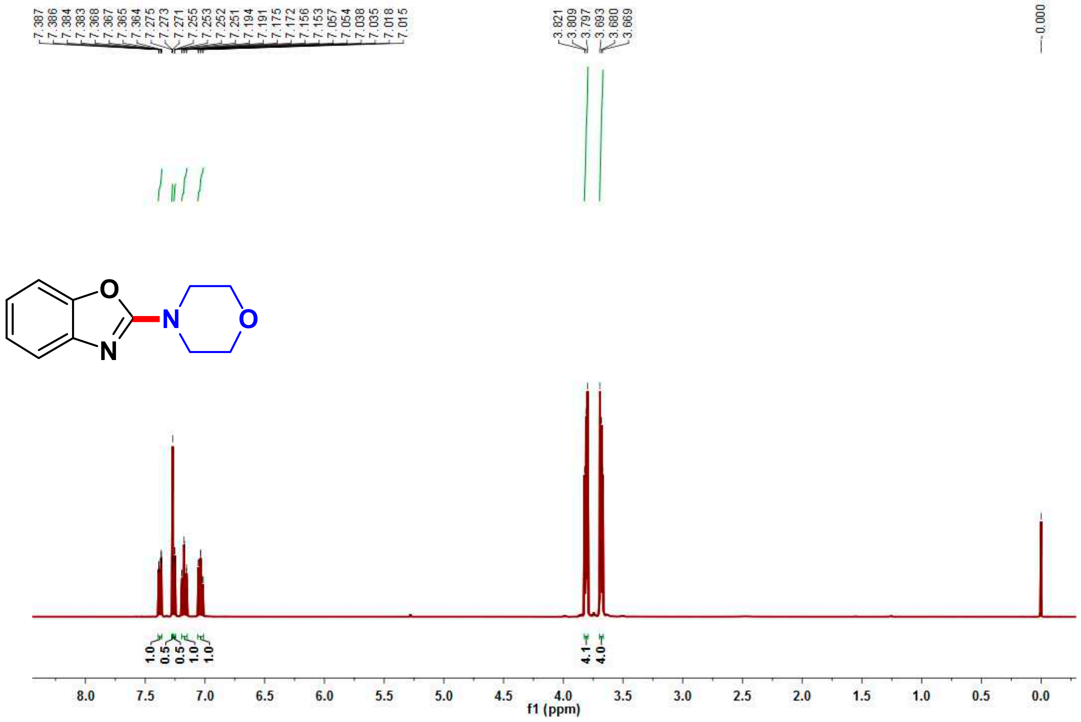

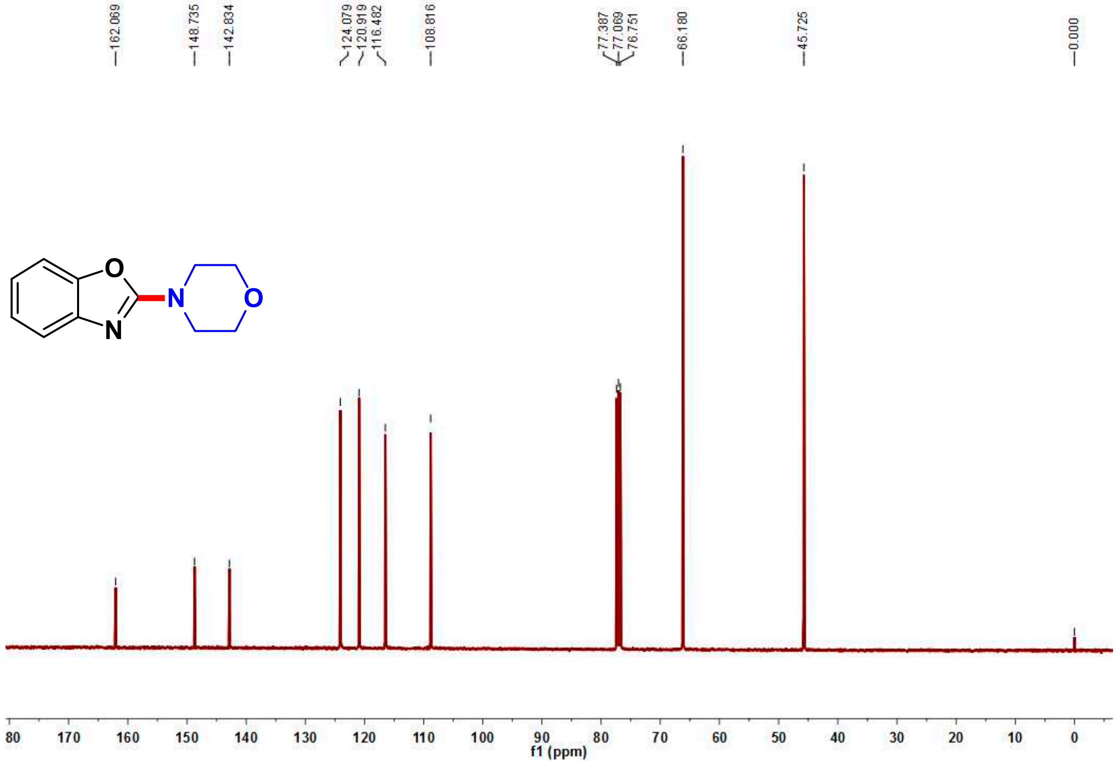

2-(Piperidin-1-yl)benzo[*d*]oxazole (3b)

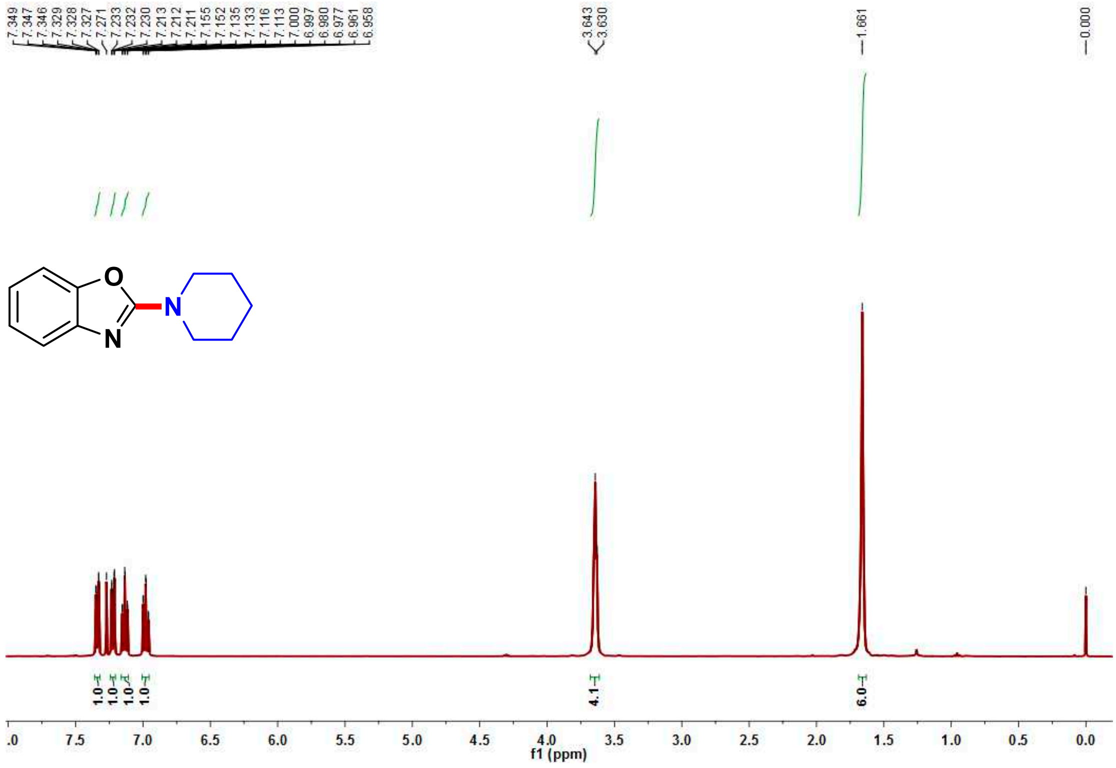

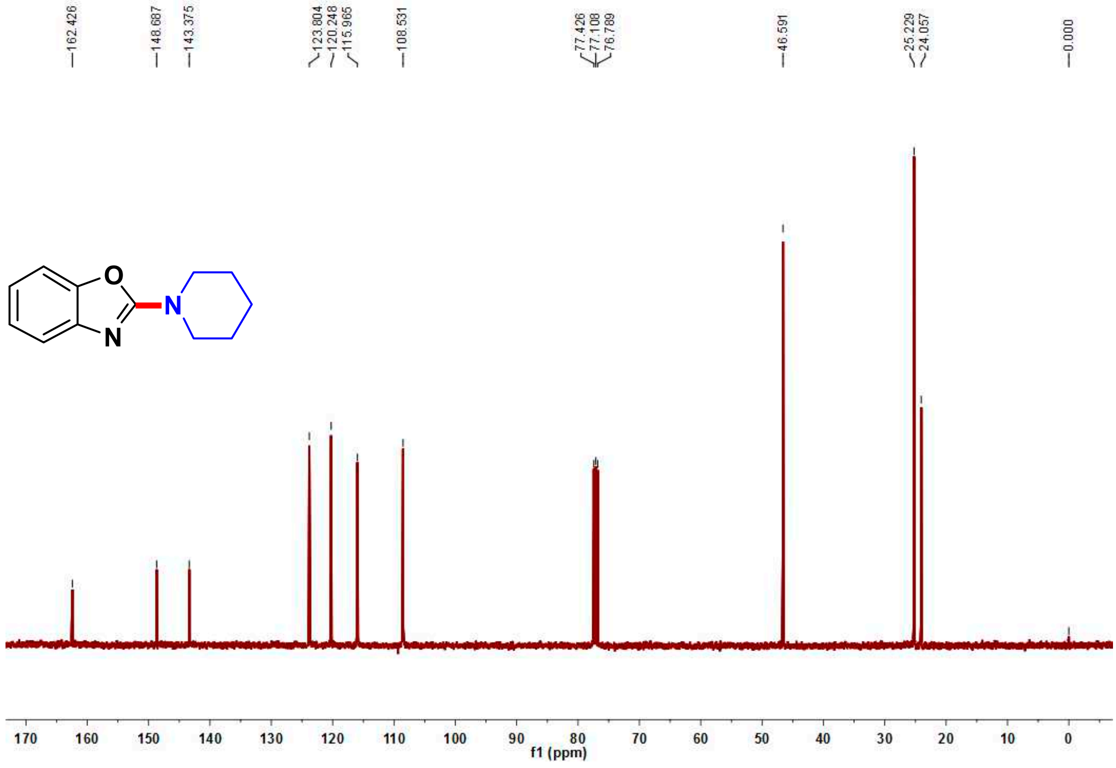

2-Thiomorpholinobenzo[*d*]oxazole (3c)

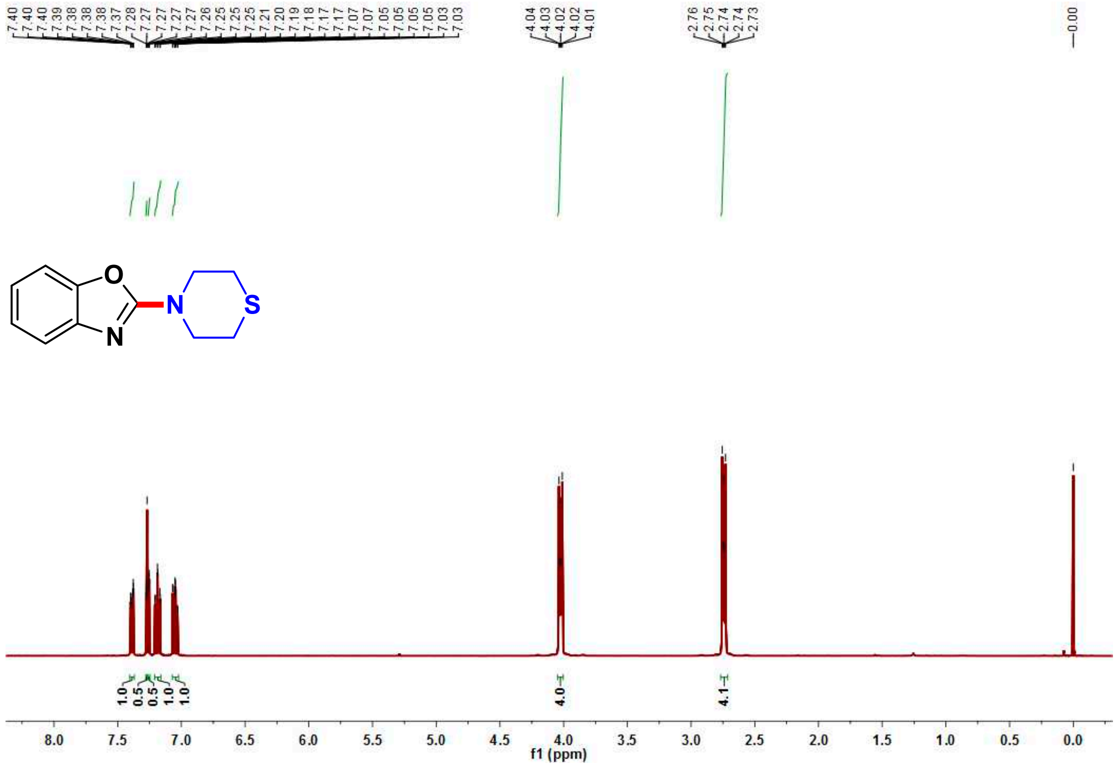

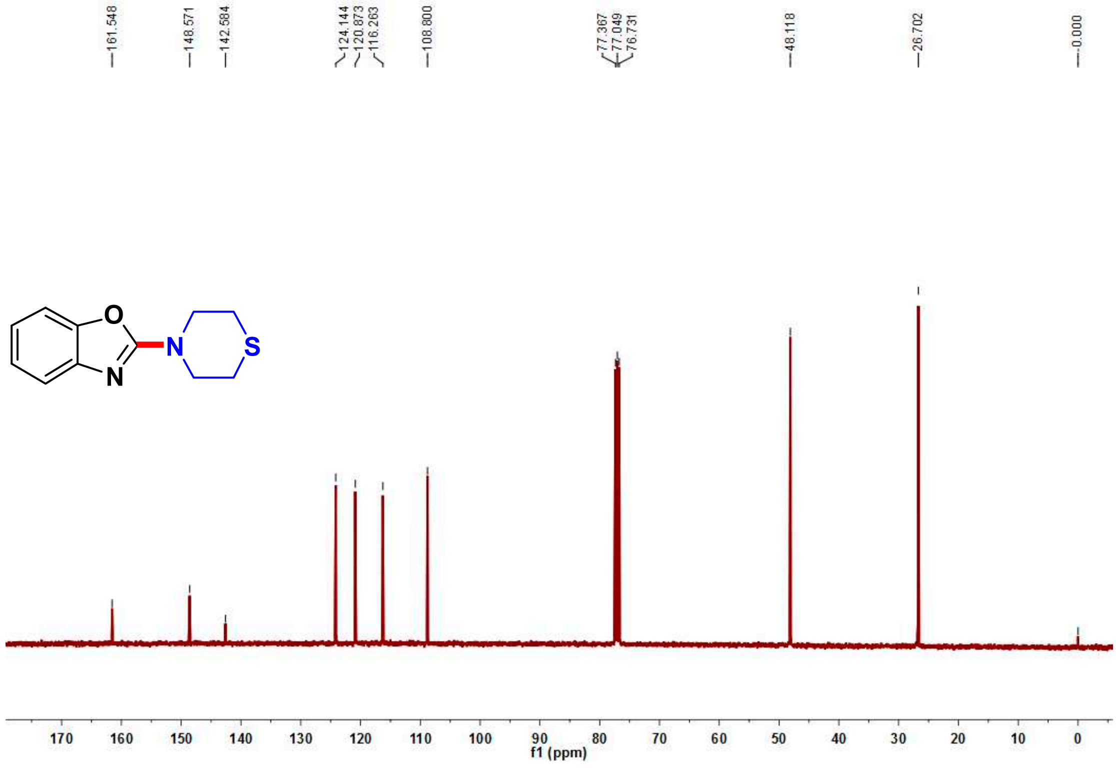

2-(3-Methylpiperidin-1-yl)benzo[*d*]oxazole (3d)

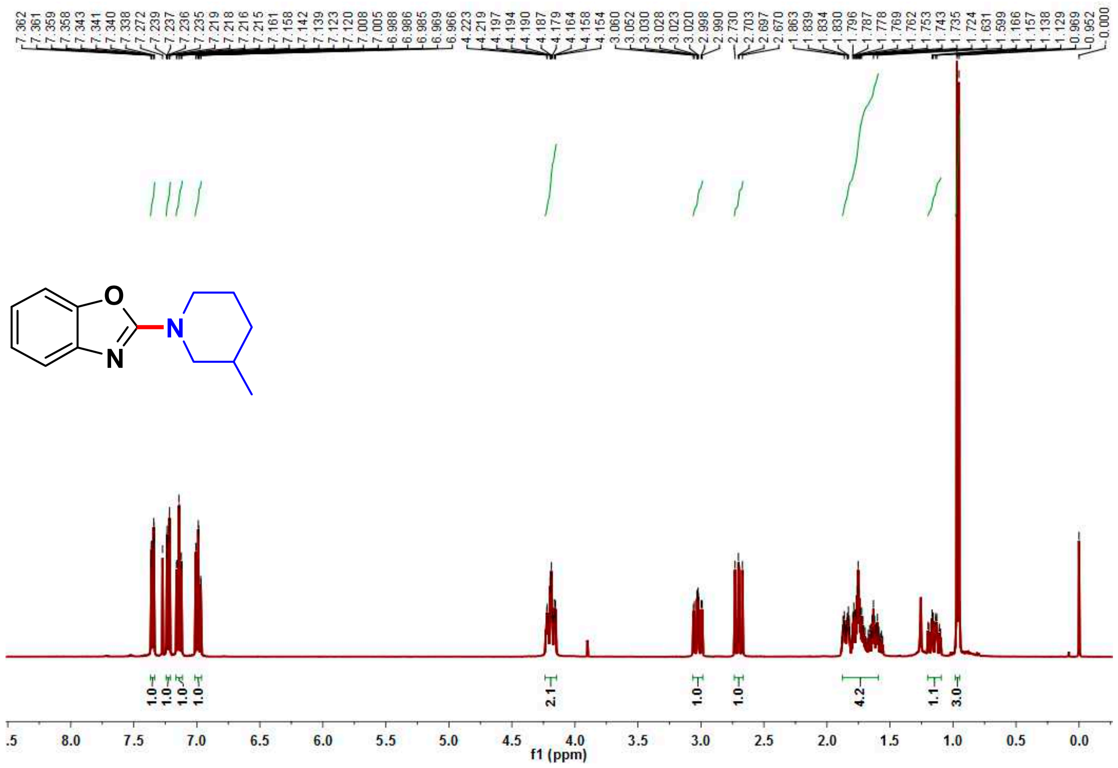

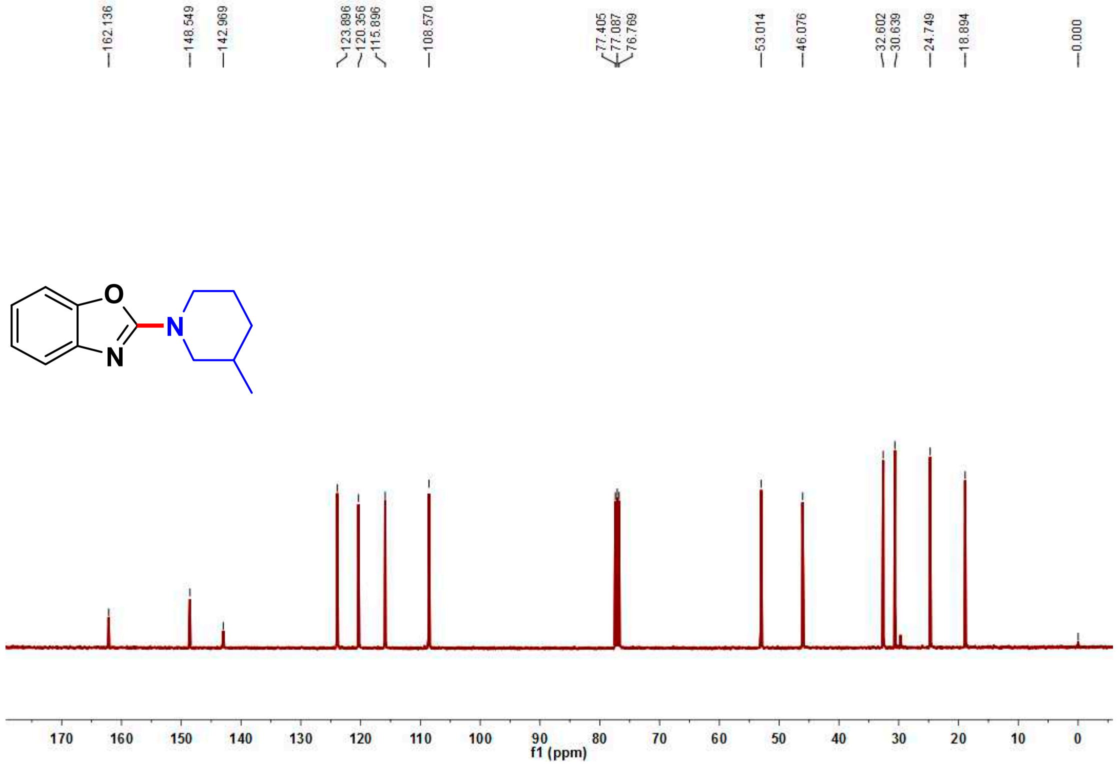

2-(4-Methylpiperazin-1-yl)benzo[*d*]oxazole (3e)

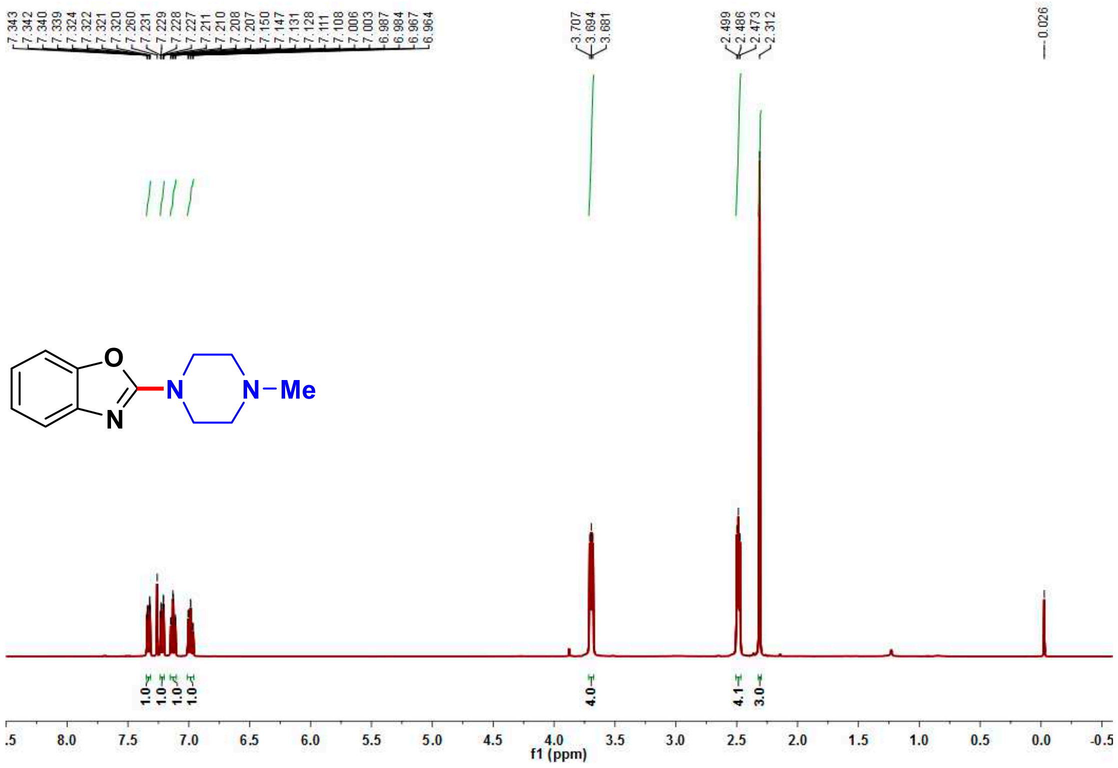

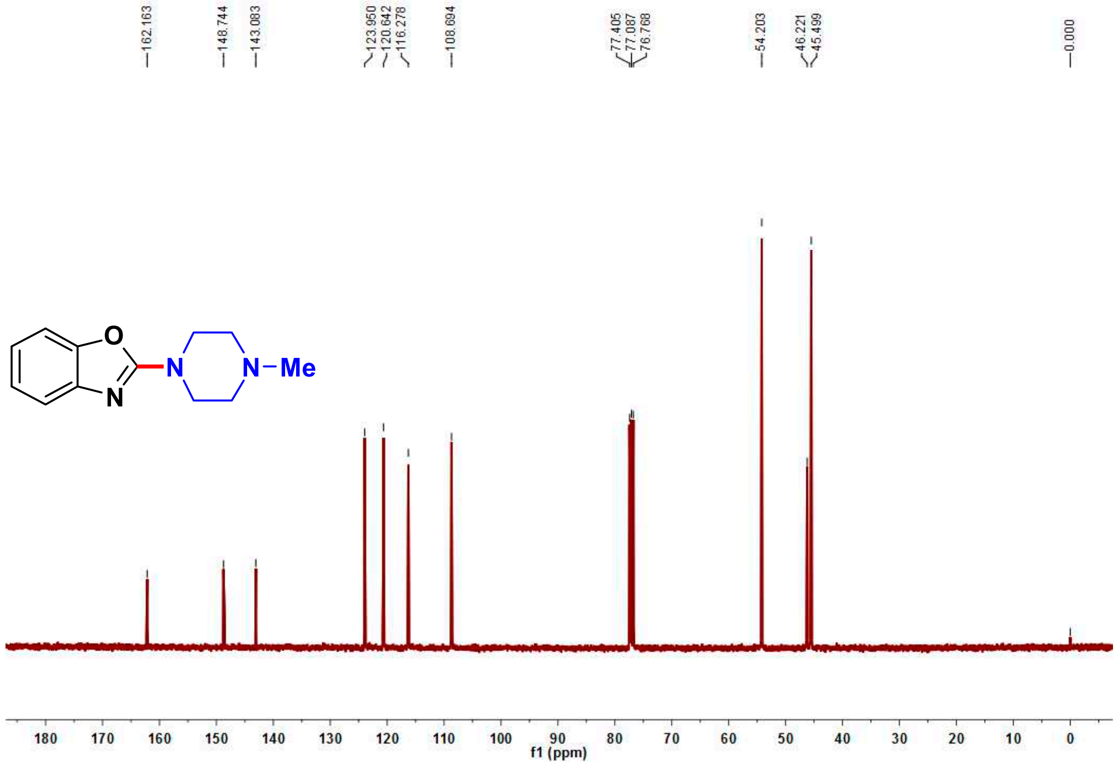

1-(4-(Benzo[*d*]oxazol-2-yl)piperazin-1-yl)ethan-1-one (3f)

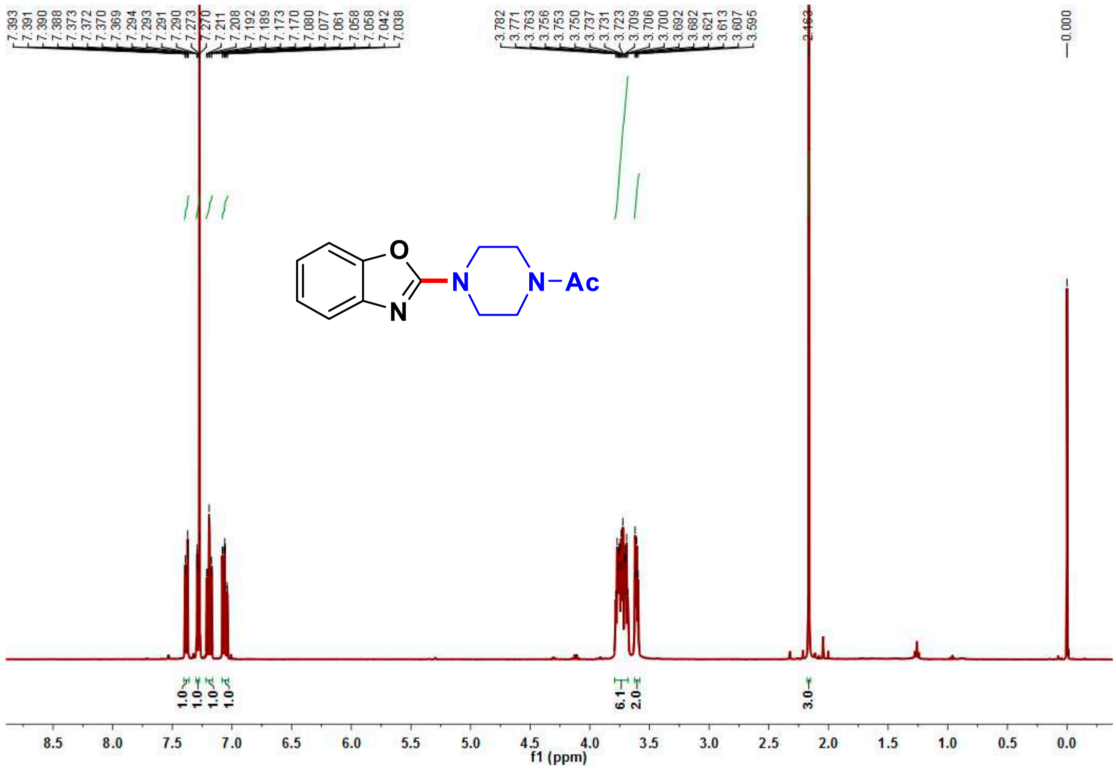

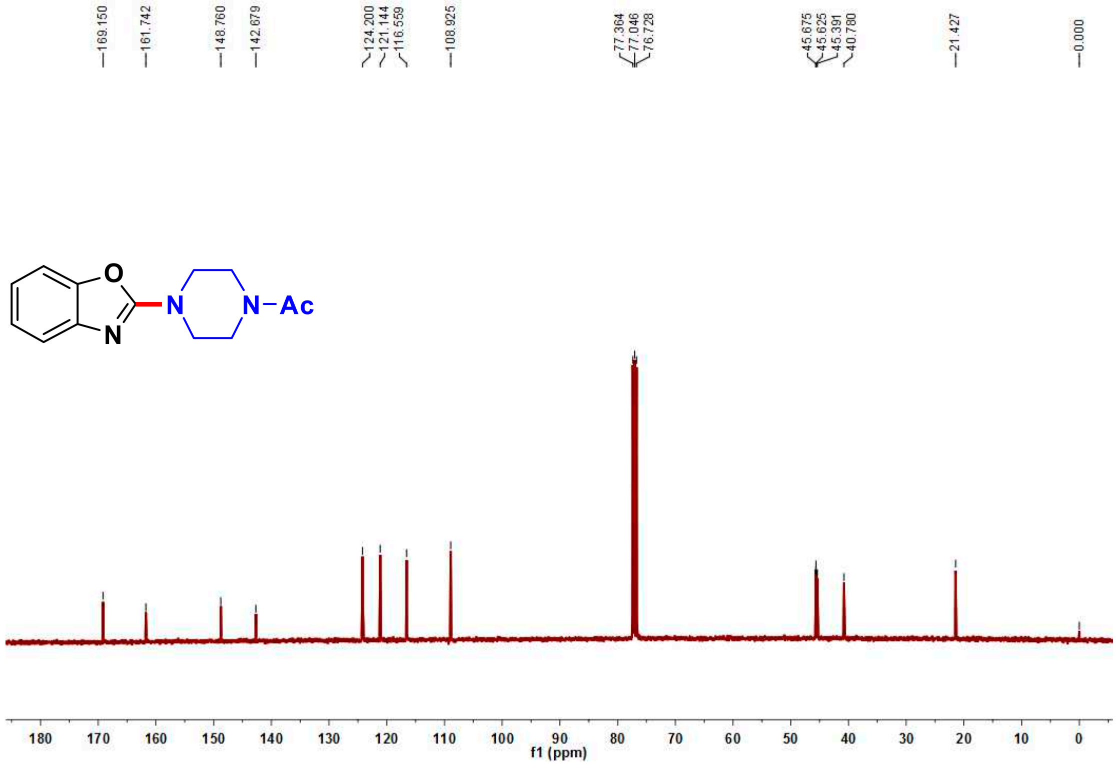

Ethyl 4-(benzo[*d*]oxazol-2-yl)piperazine-1-carboxylate (3g)

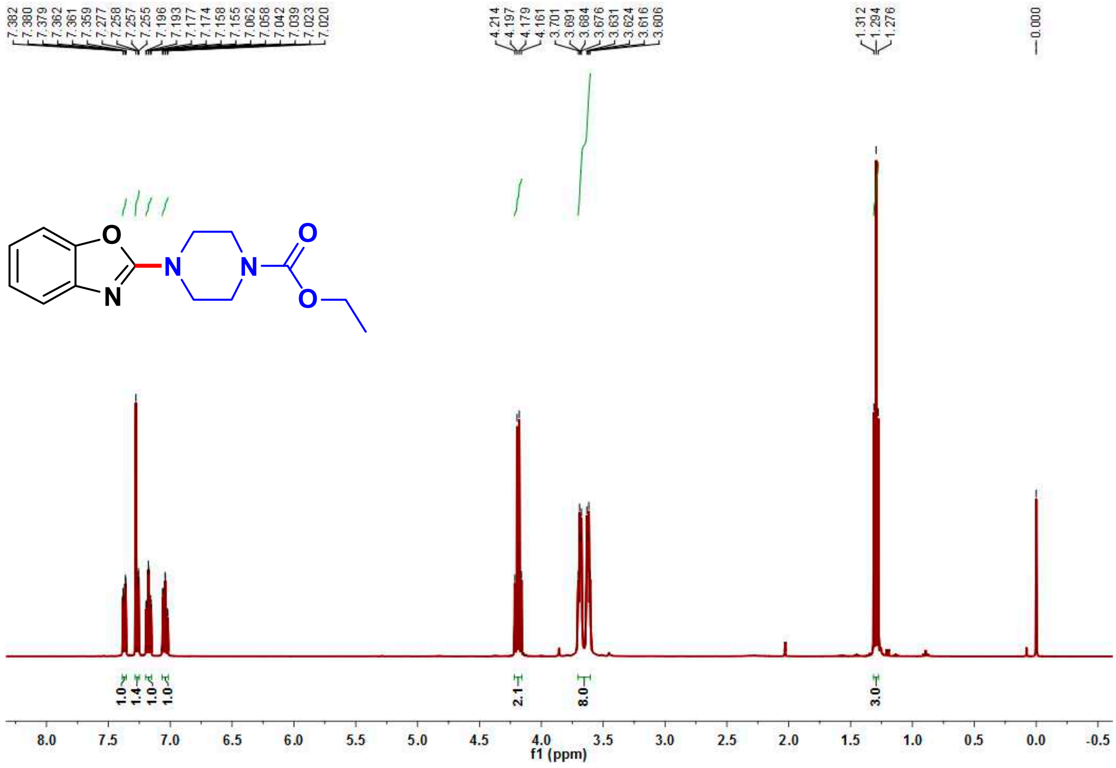

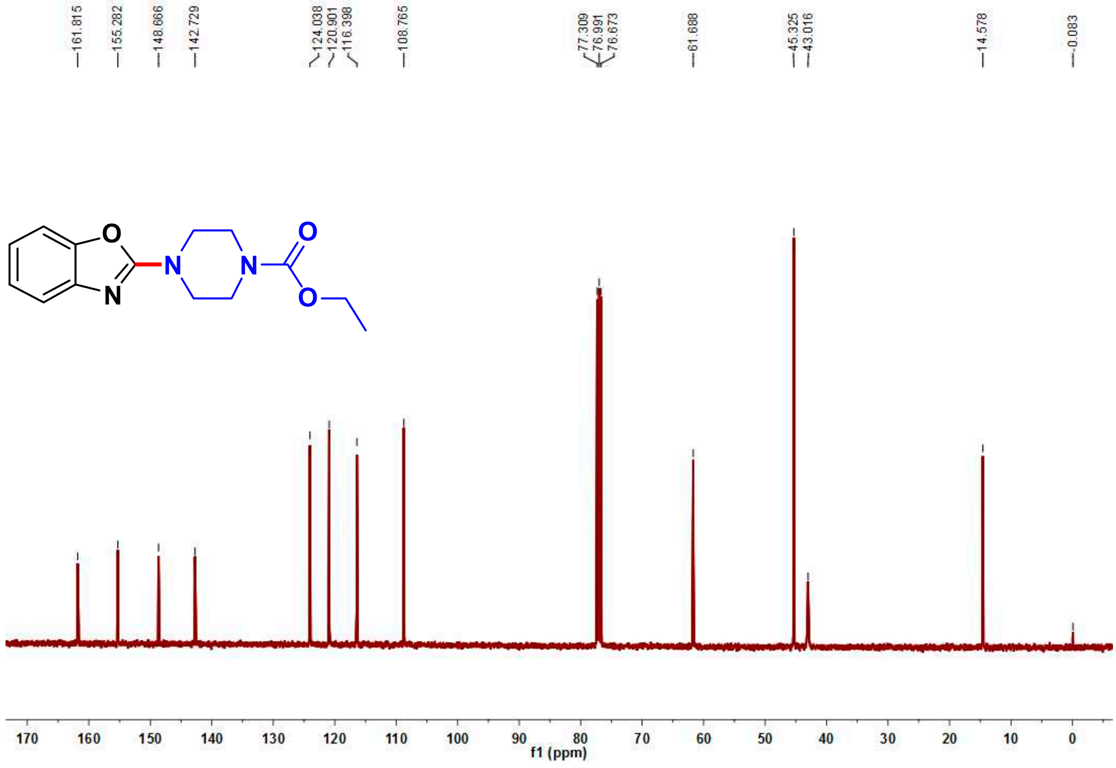

2-(4-Phenylpiperazin-1-yl)benzo[d]oxazole (3h)

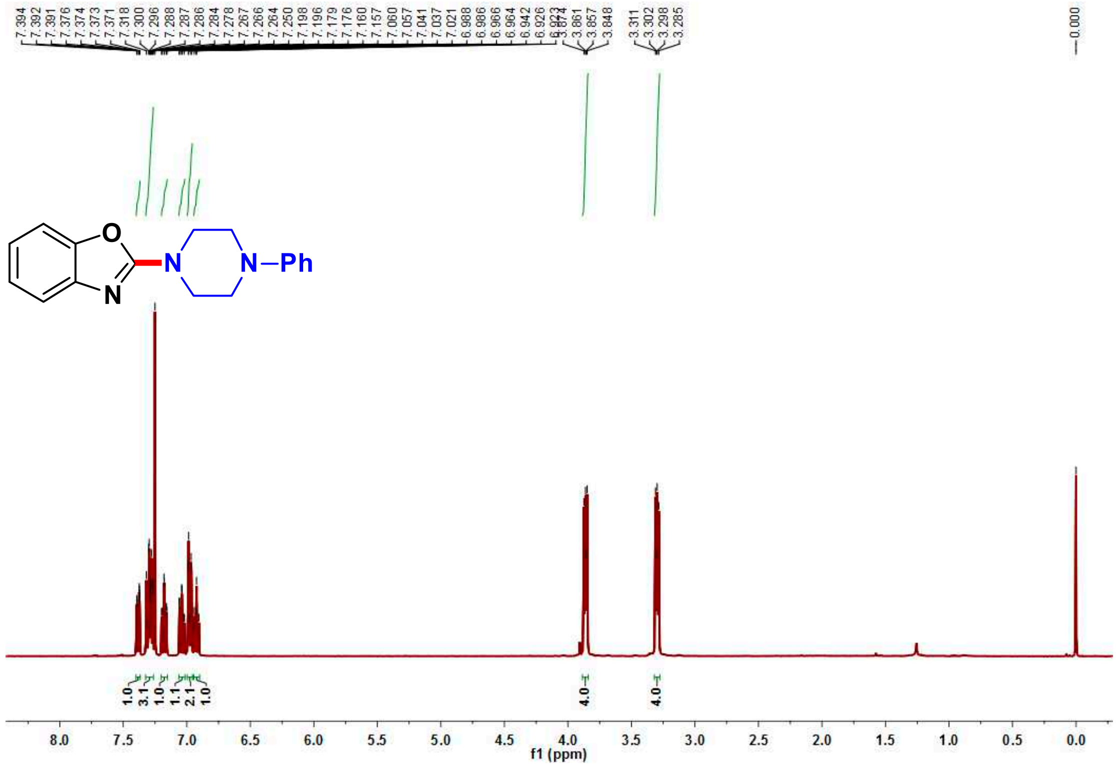

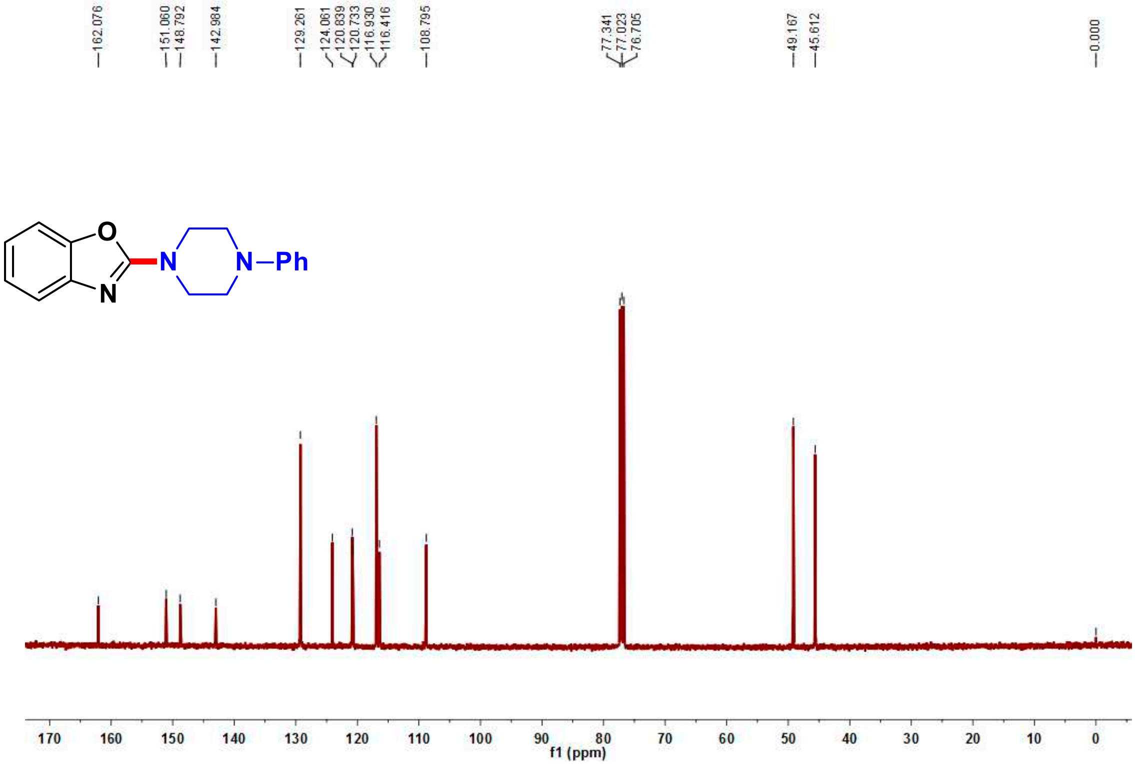

***Tert*-butyl 4-(benzo[*d*]oxazol-2-yl)piperazine-1-carboxylate (3i)**

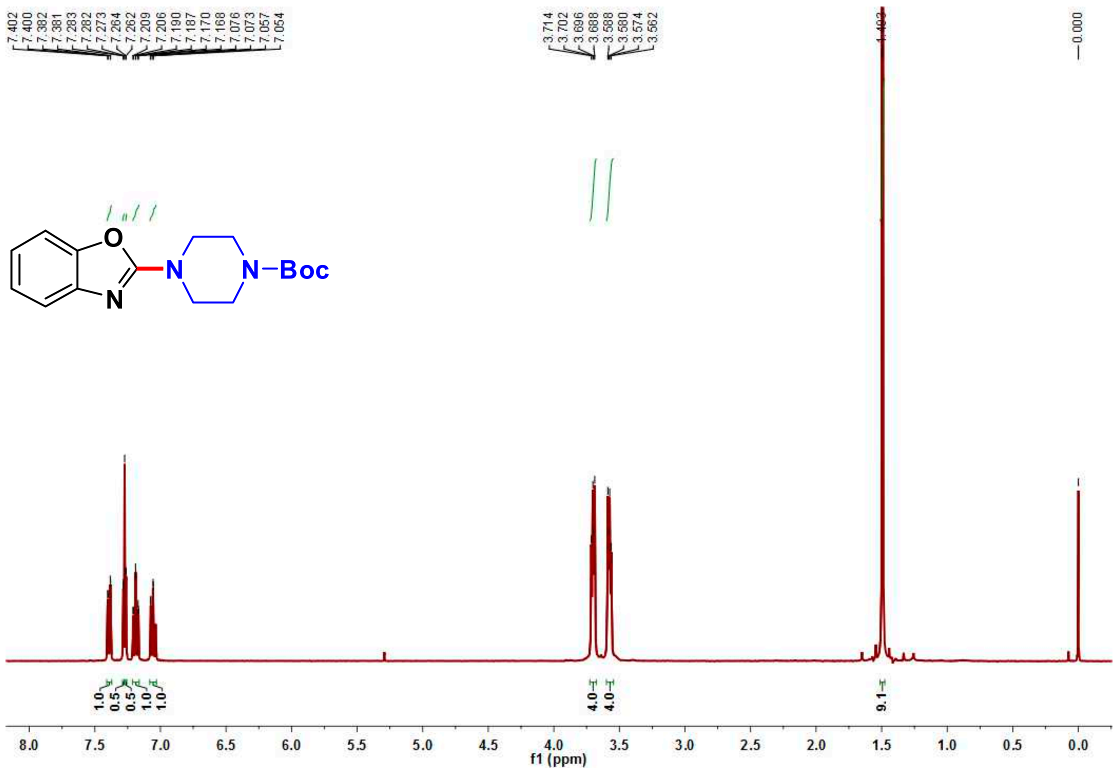

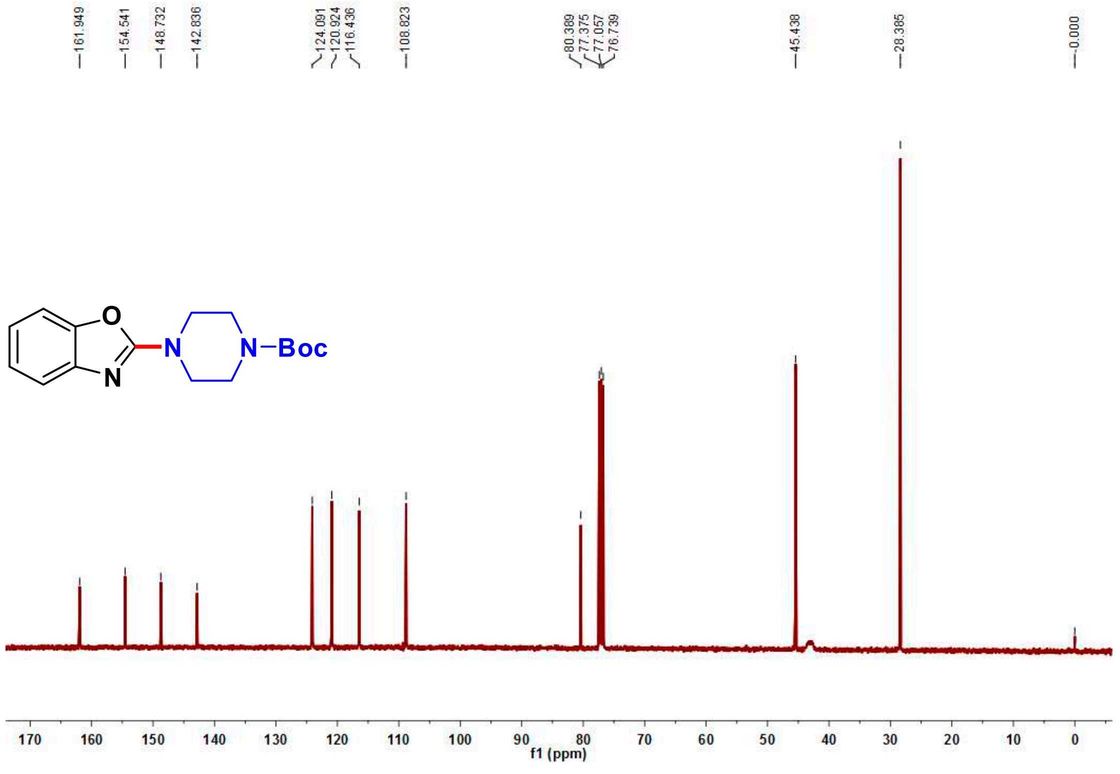

2-(3,4-Dihydroisoquinolin-2(1*H*)-yl)benzo[*d*]oxazole (3j)

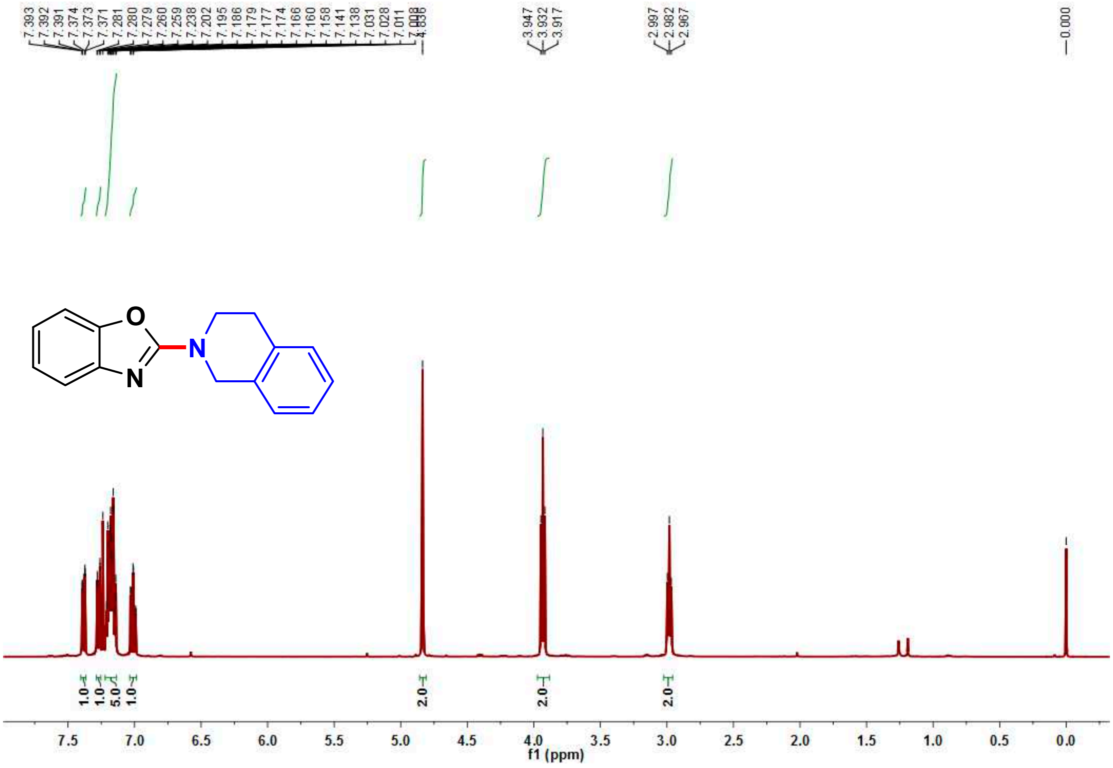

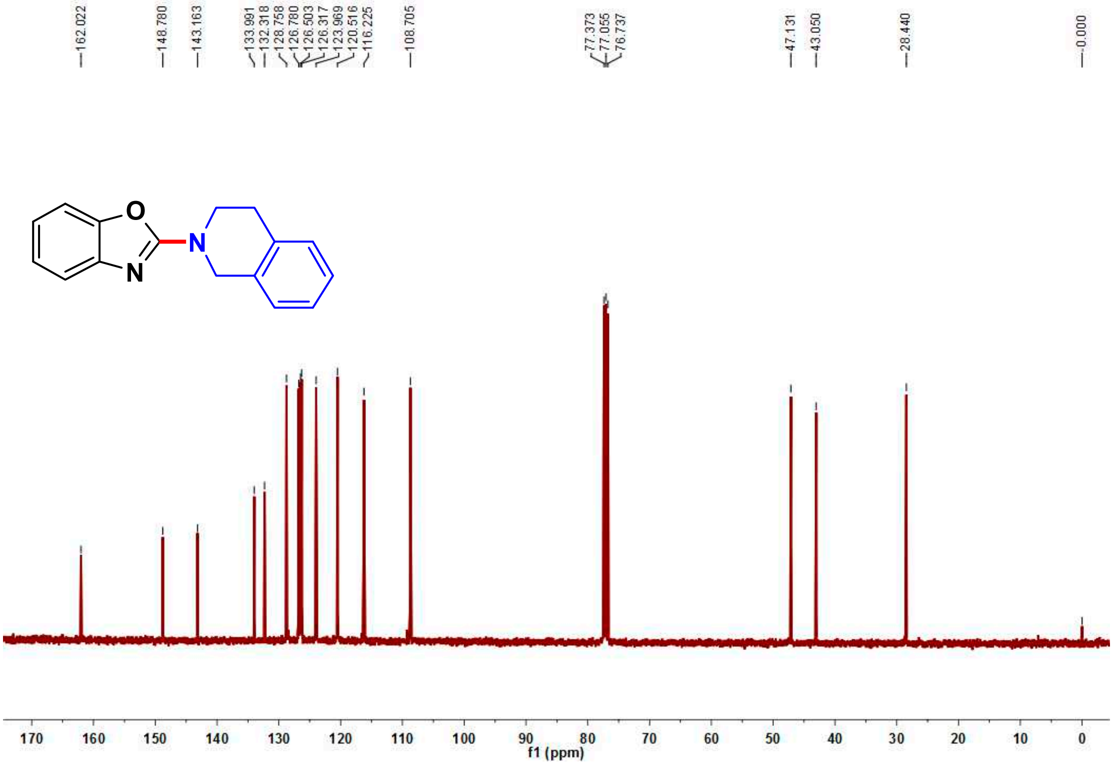

N,*N*-Diethylbenzo[*d*]oxazol-2-amine (3k)

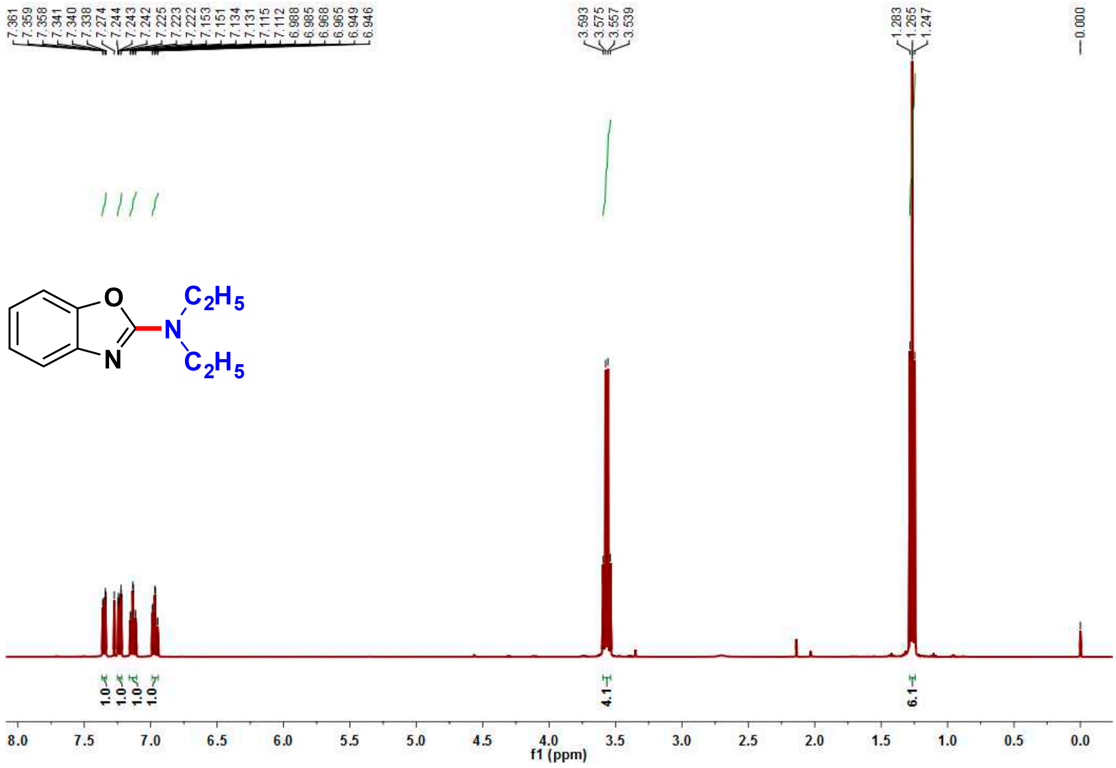

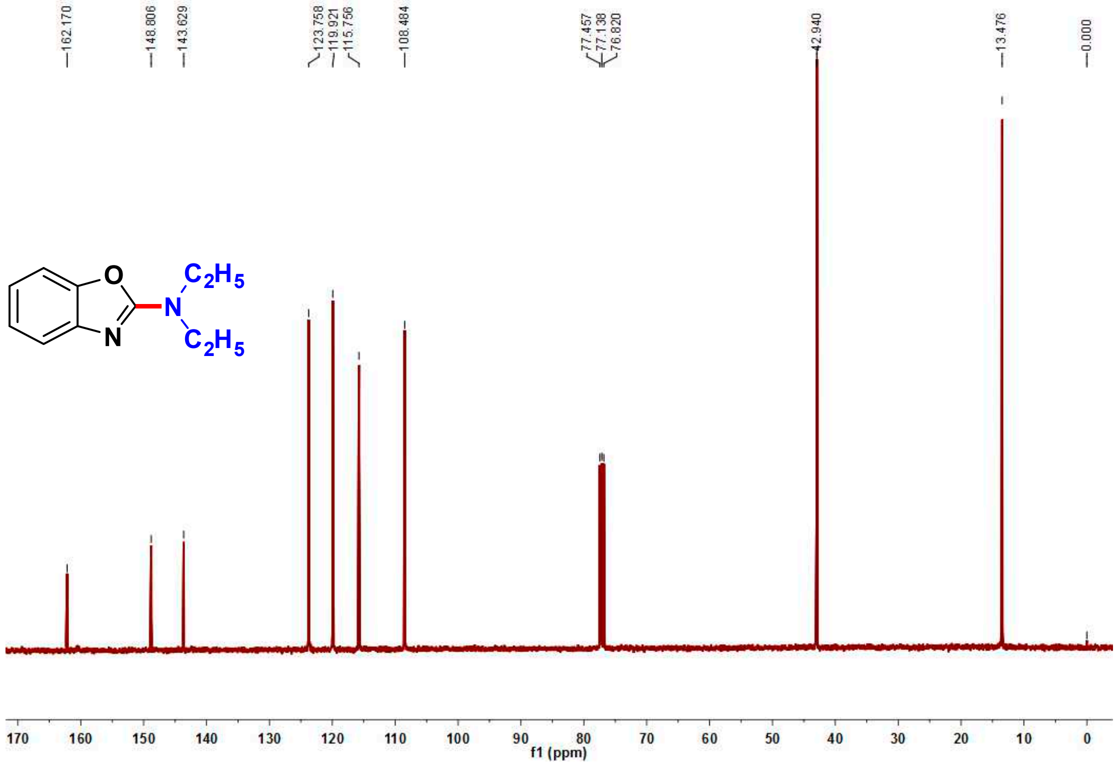

N,*N*-Dibenzylbenzo[*d*]oxazol-2-amine (3l)

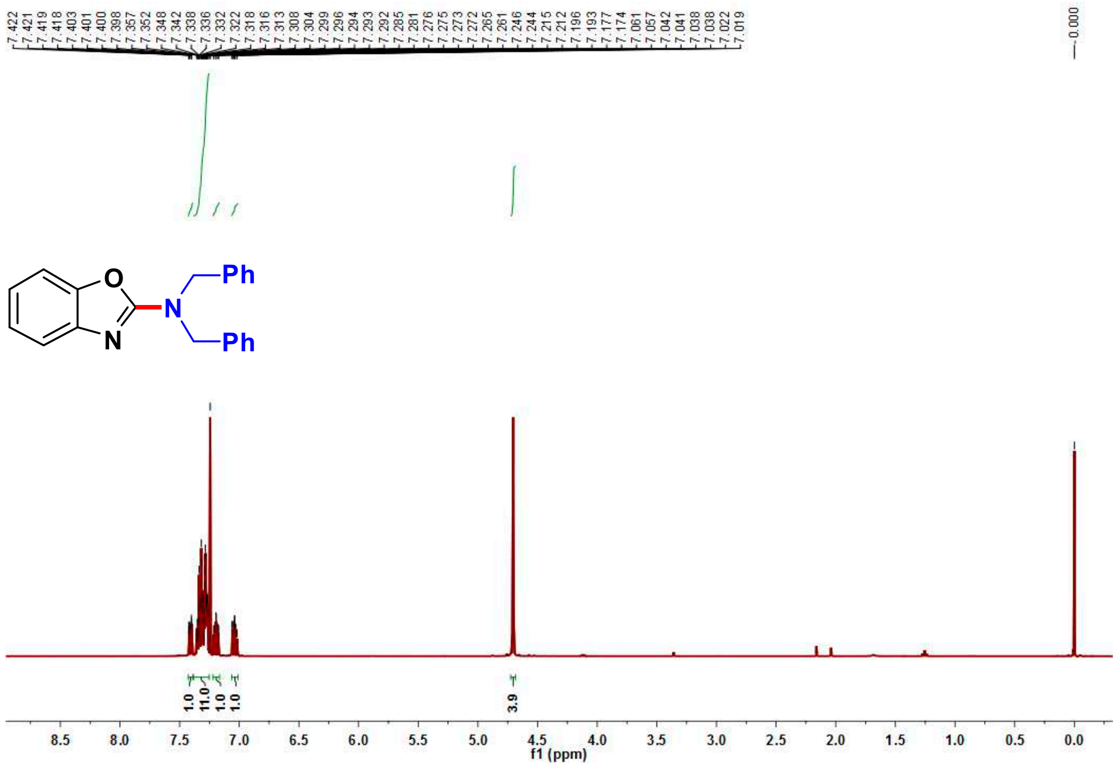

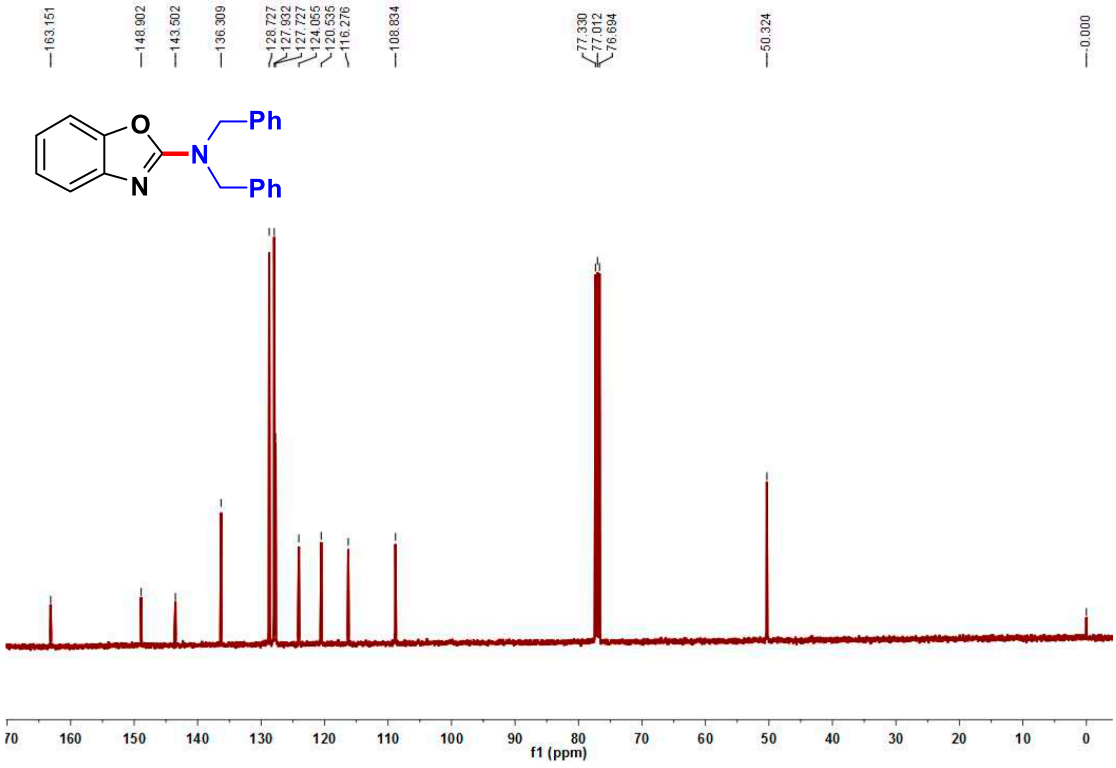

N,*N*-Diallylbenzo[*d*]oxazol-2-amine (3m)

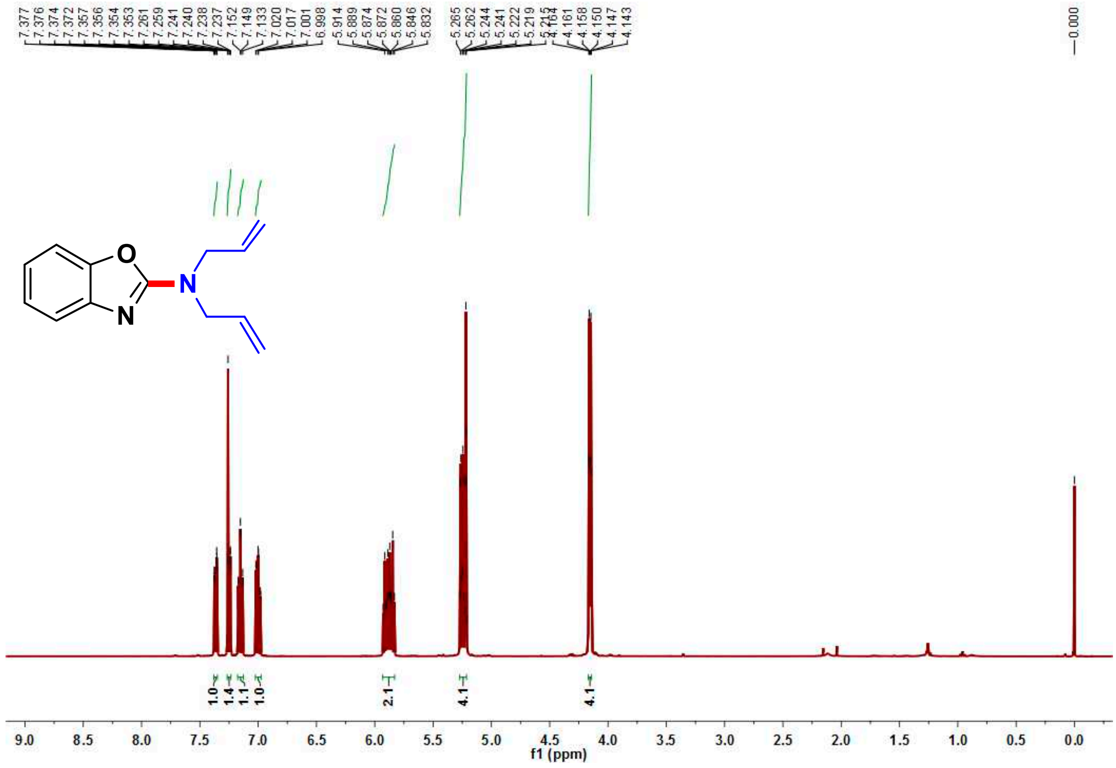

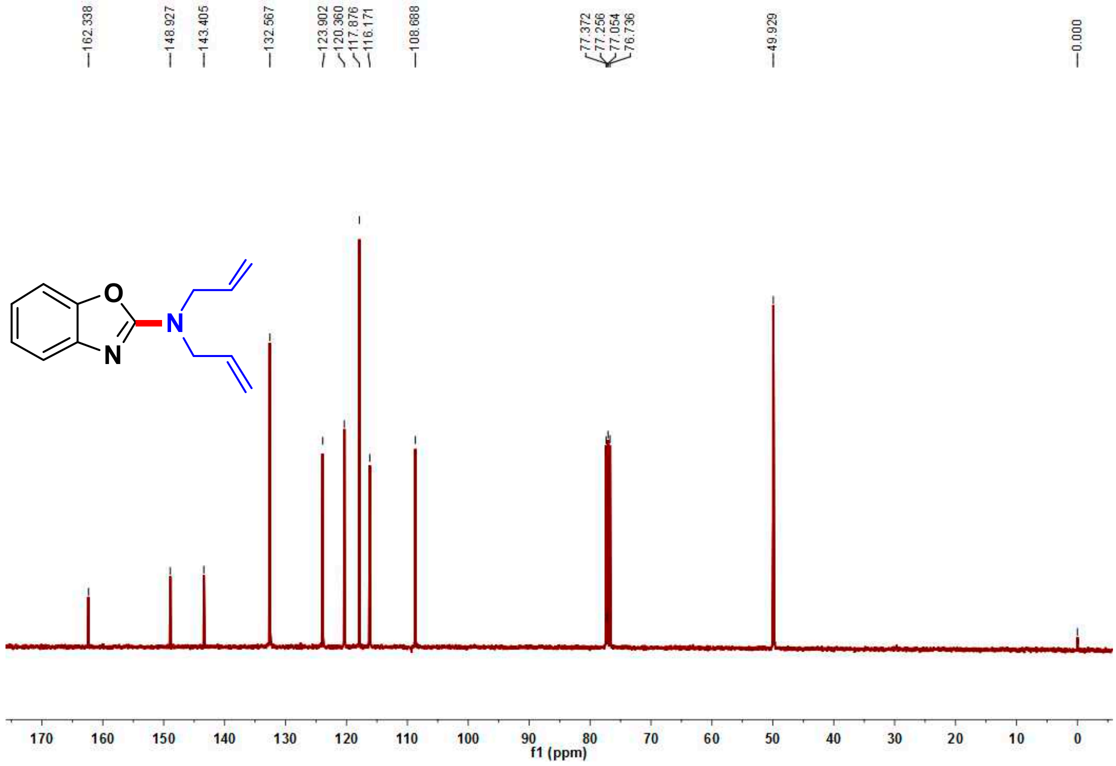

5-Methyl-2-morpholinobenzo[*d*]oxazole (4a)

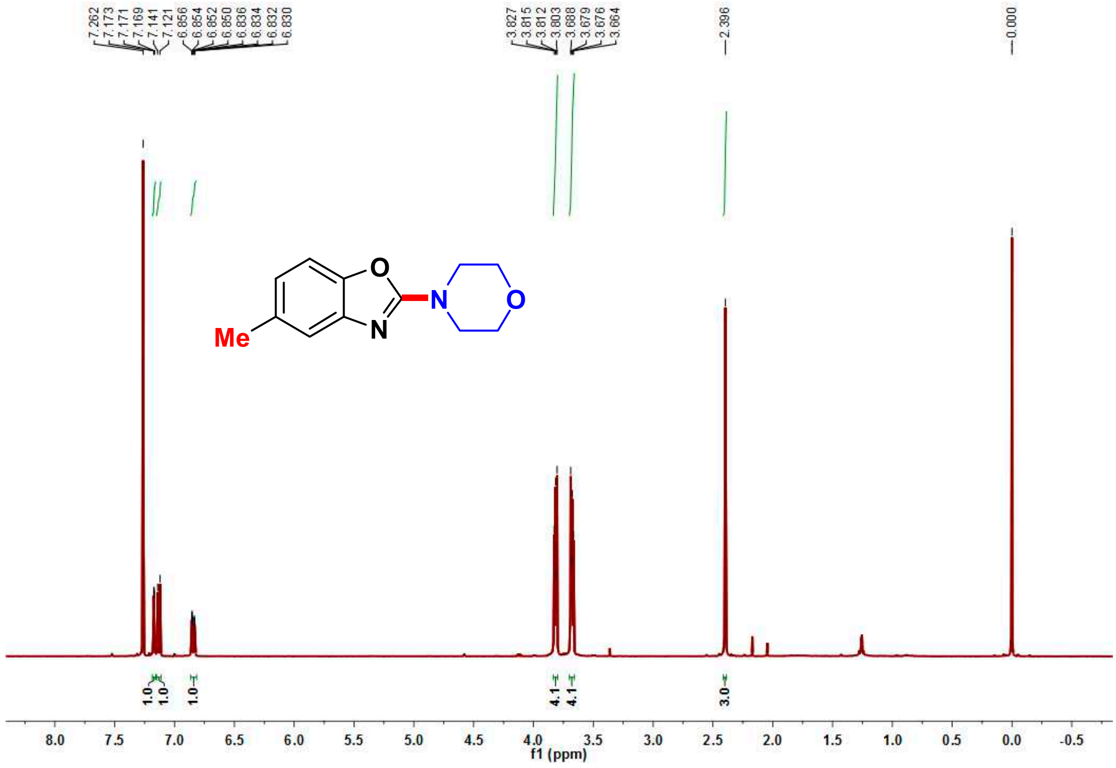

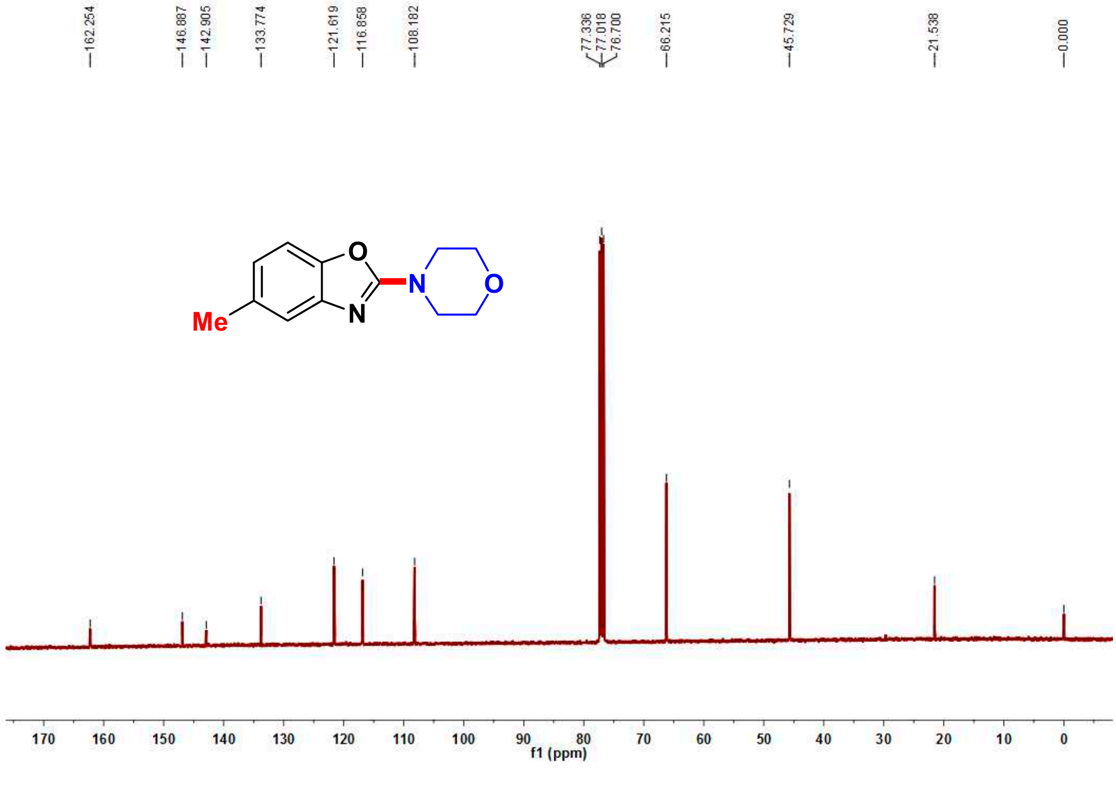

6-Methyl-2-morpholinobenzo[*d*]oxazole (4b)

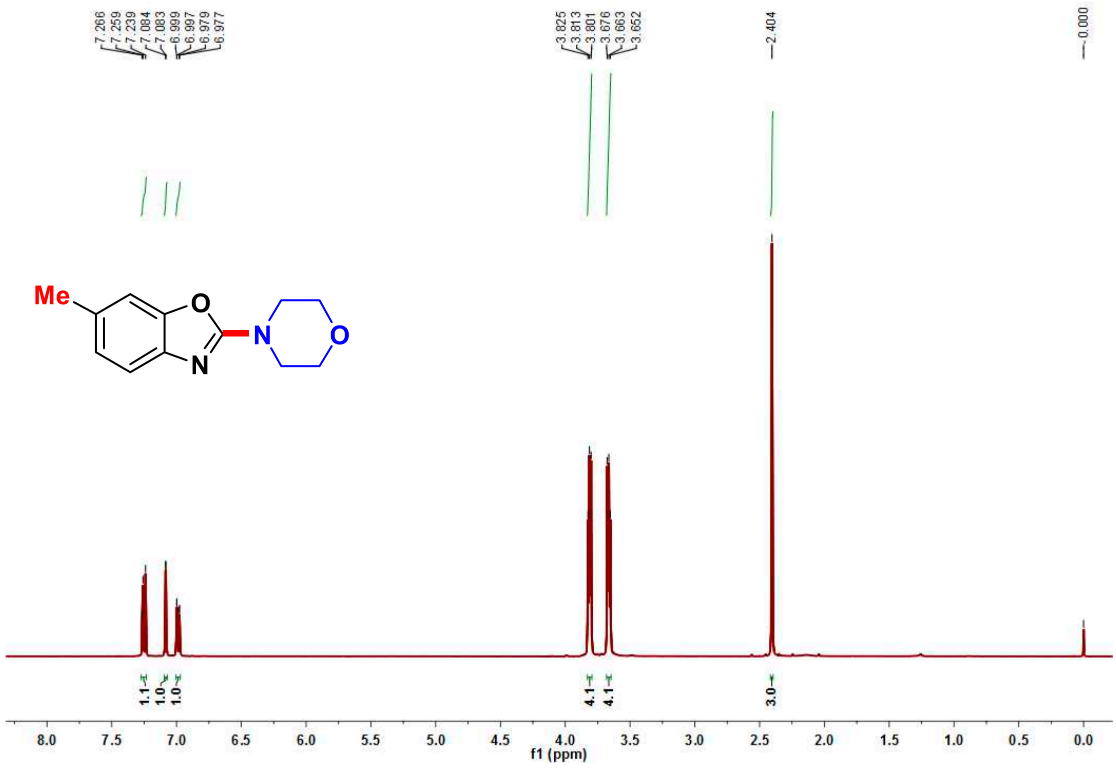

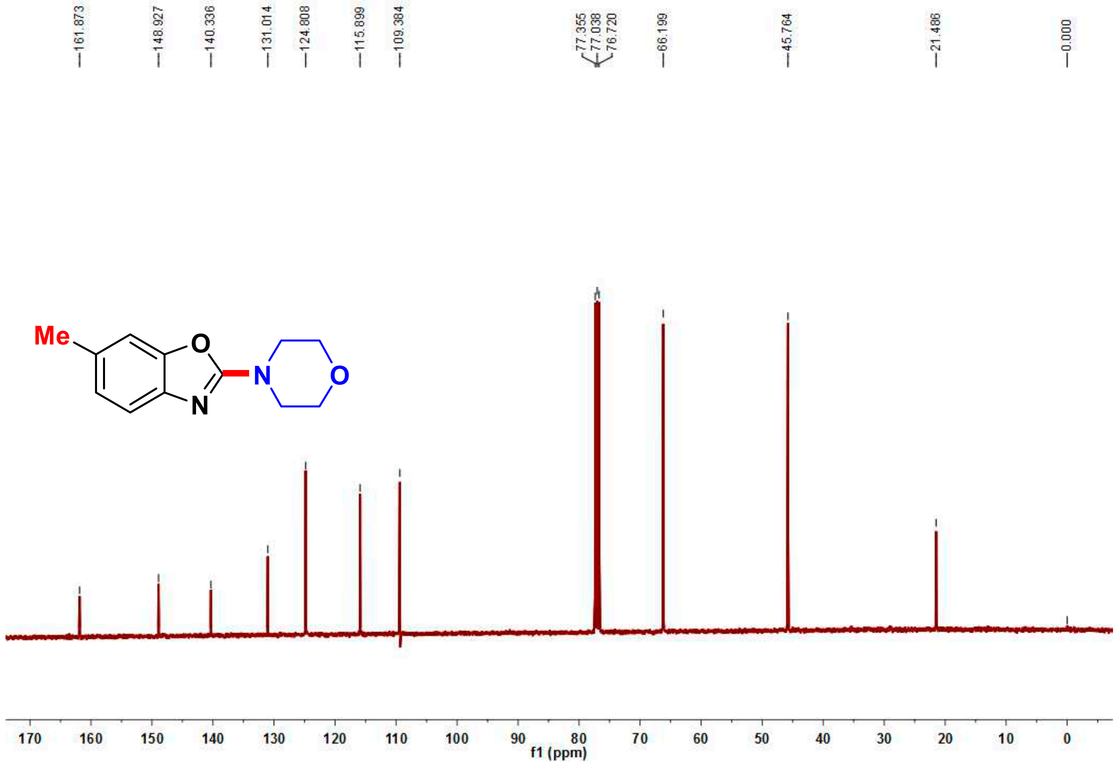

5-Chloro-2-morpholinobenzo[*d*]oxazole (4c)

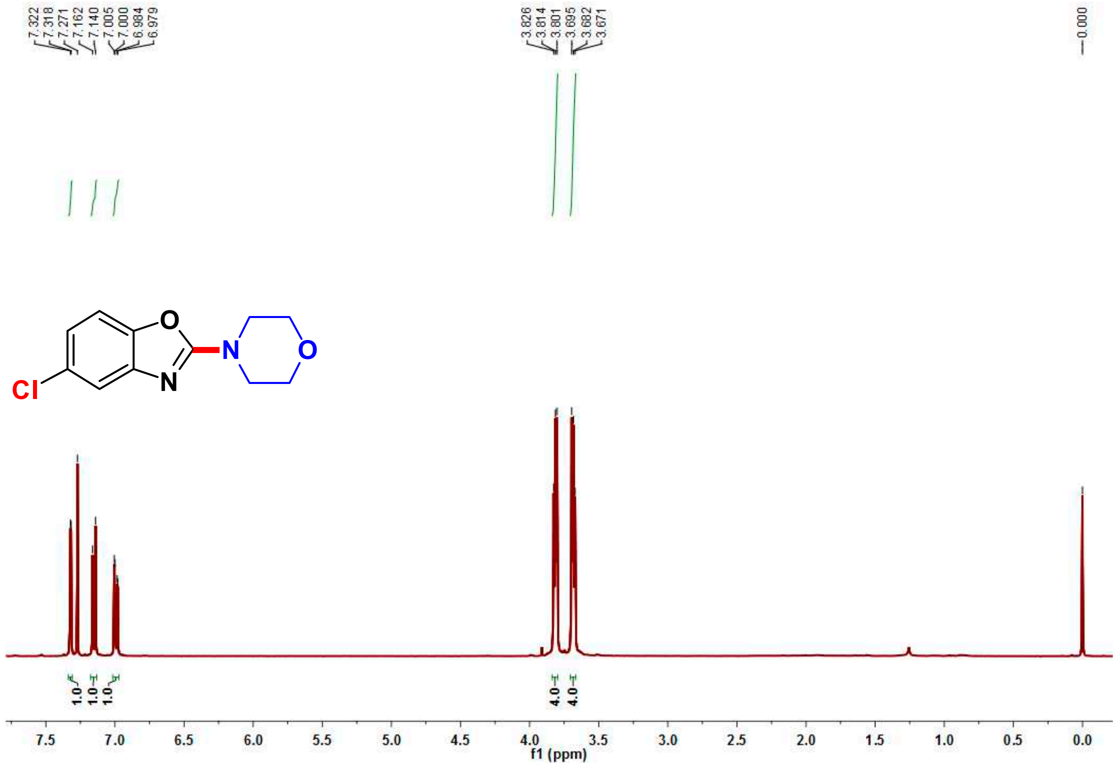

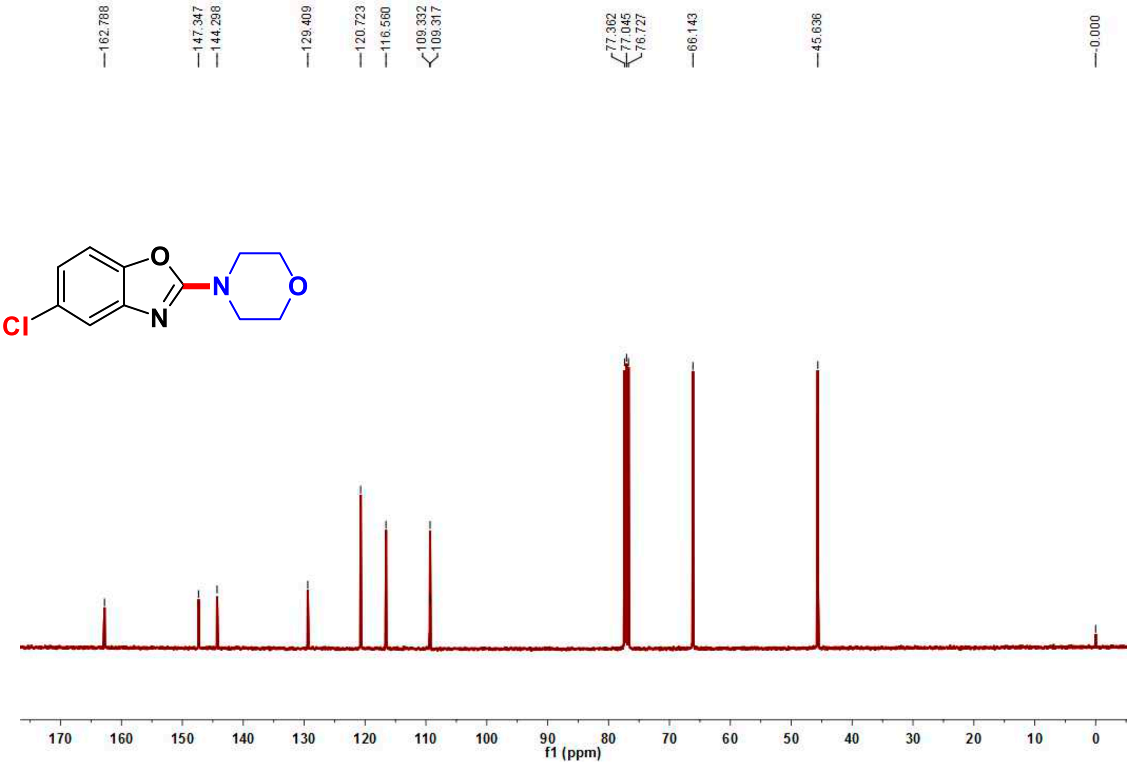

5-Bromo-2-morpholinobenzo[*d*]oxazole (4d)

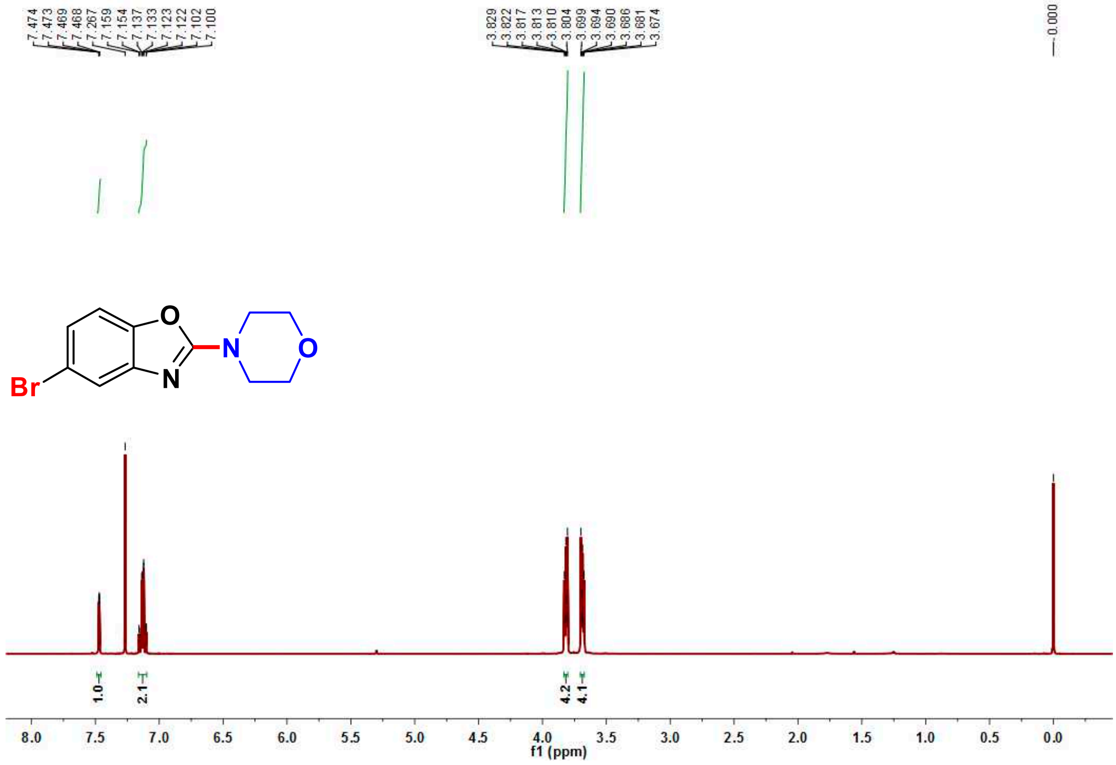

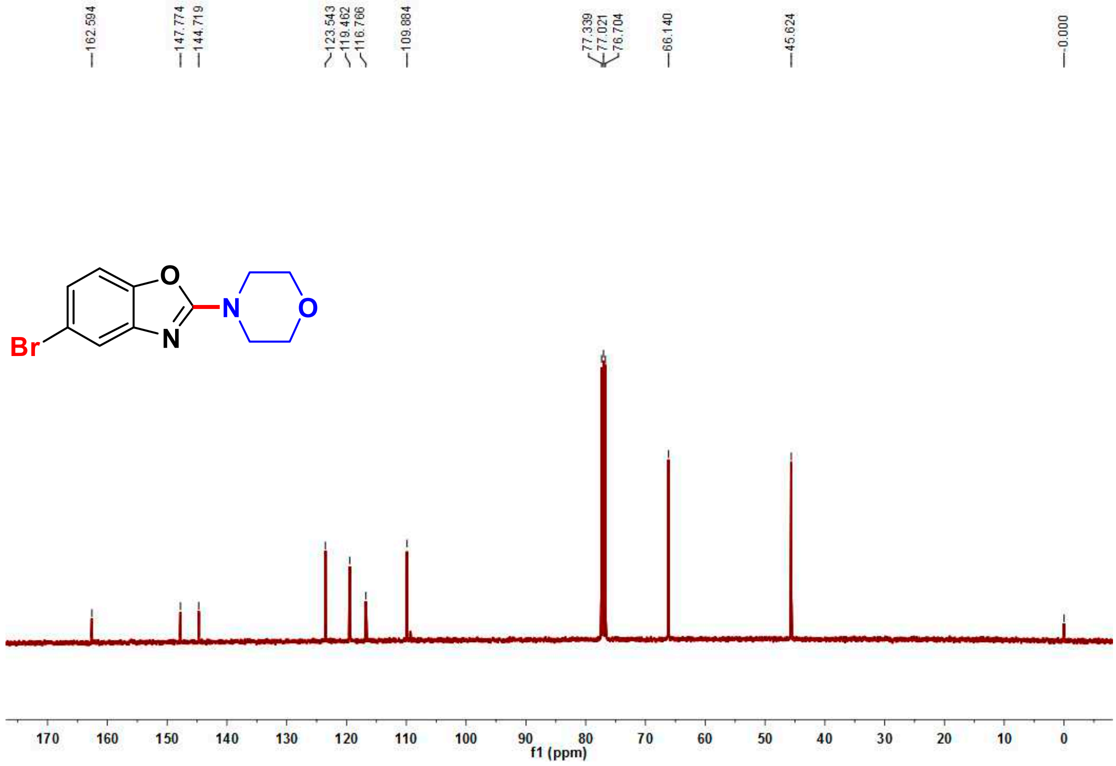

N,*N*-Diallyl-5-methylbenzo[*d*]oxazol-2-amine (4e)

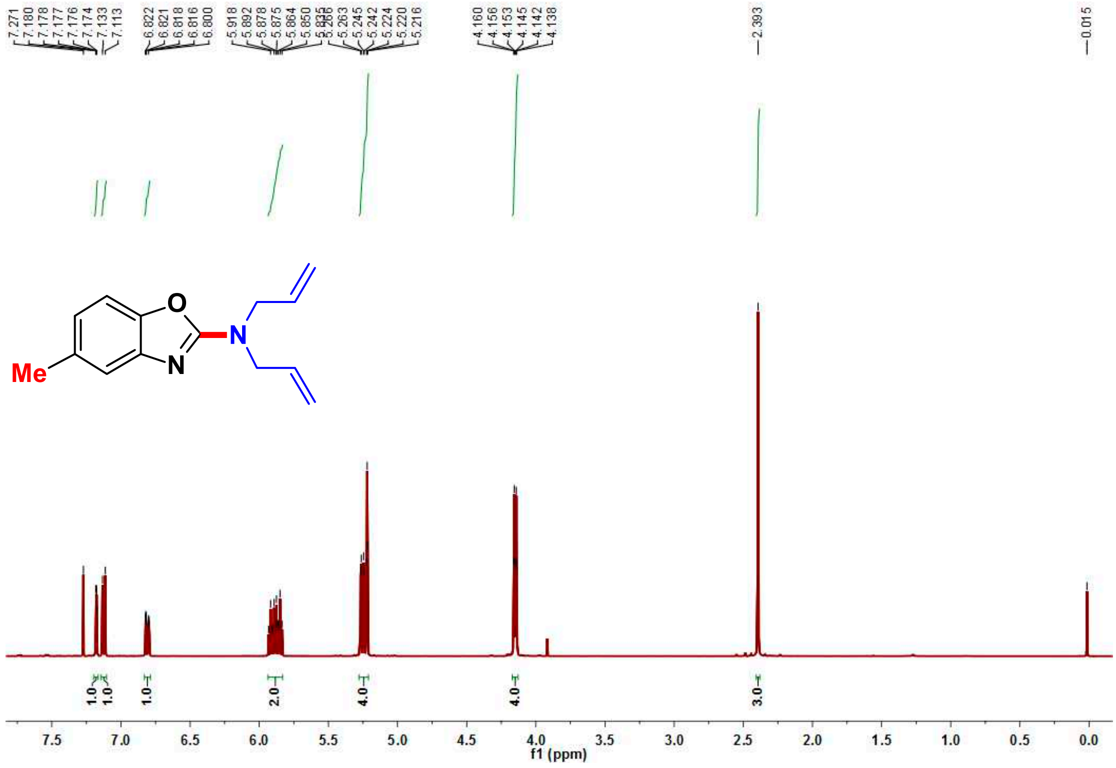

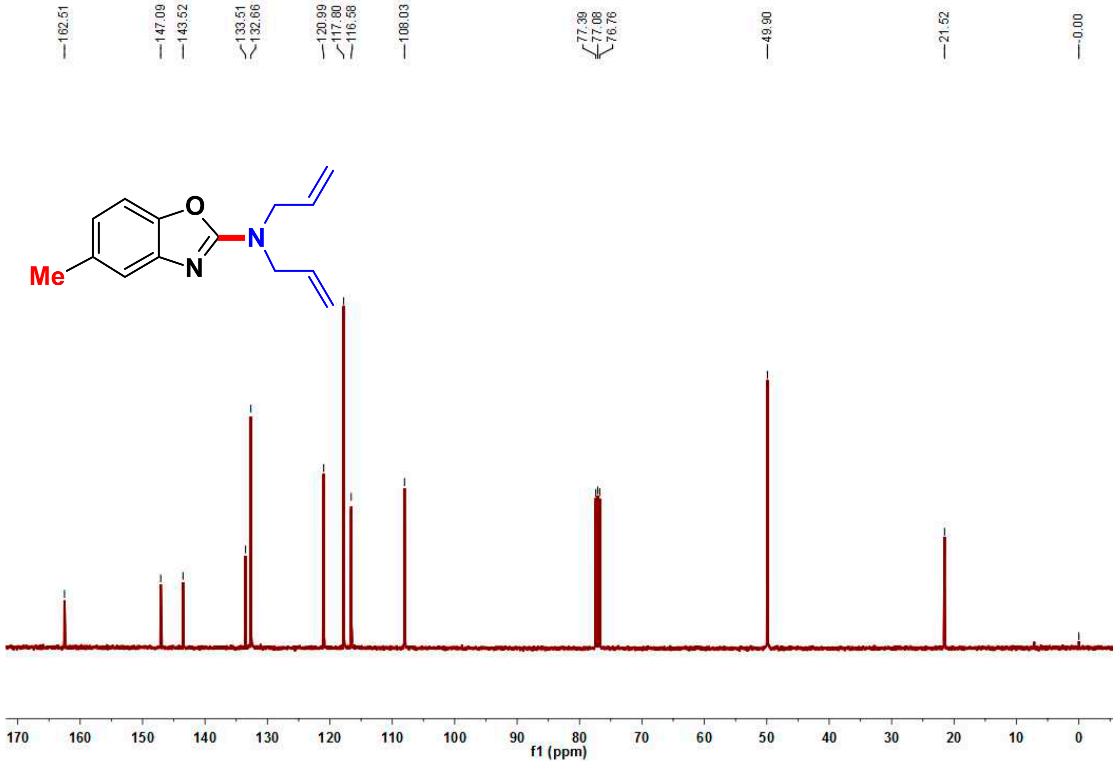

N,*N*-Dibenzyl-5-methylbenzo[*d*]oxazol-2-amine (4f)

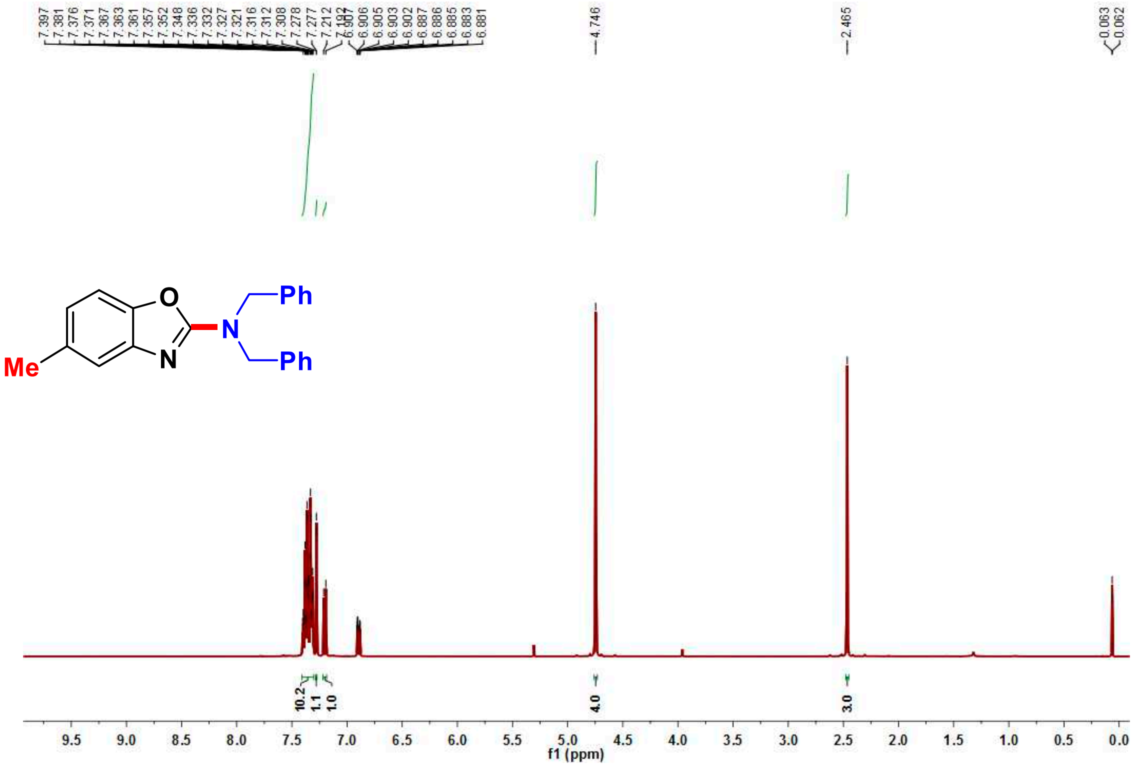

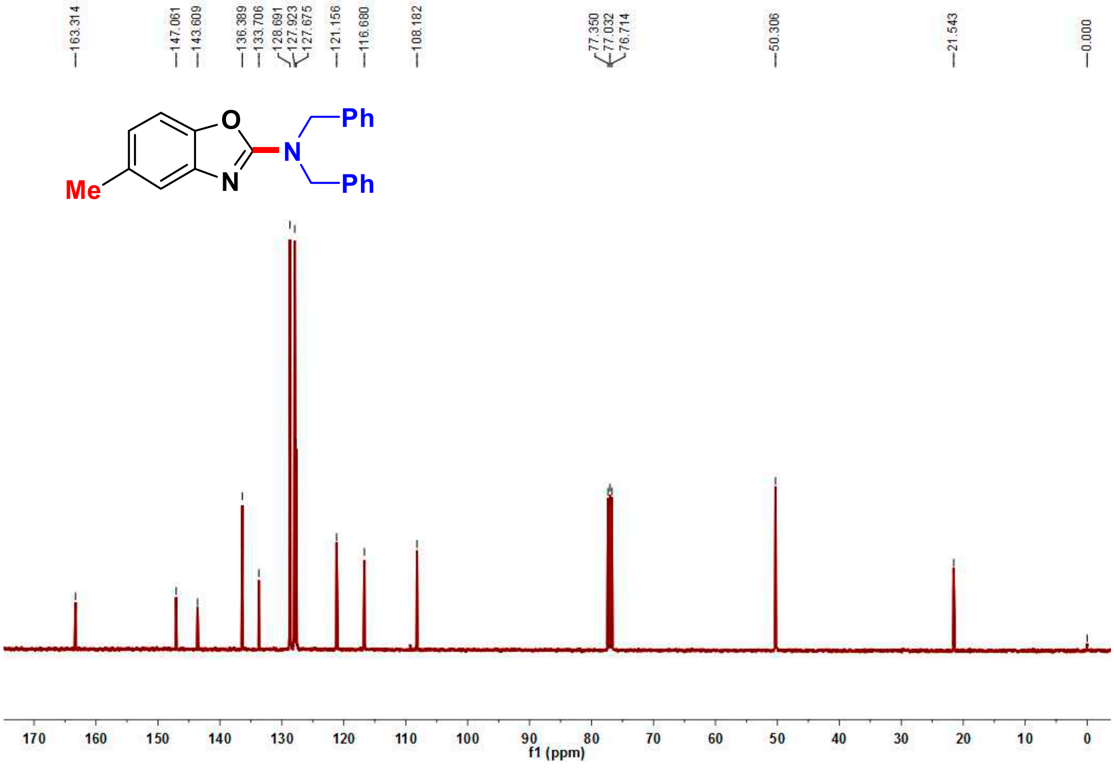

***Tert*-butyl 4-(5-methylbenzo[*d*]oxazol-2-yl)piperazine-1-carboxylate (4g)**

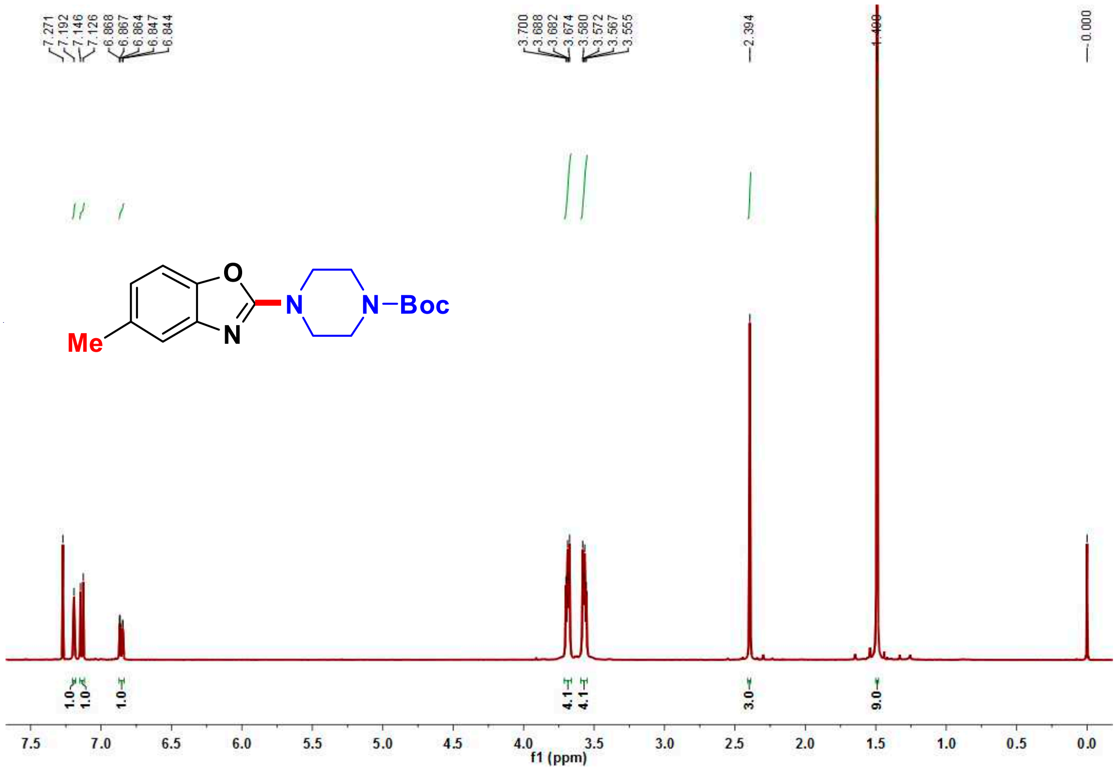

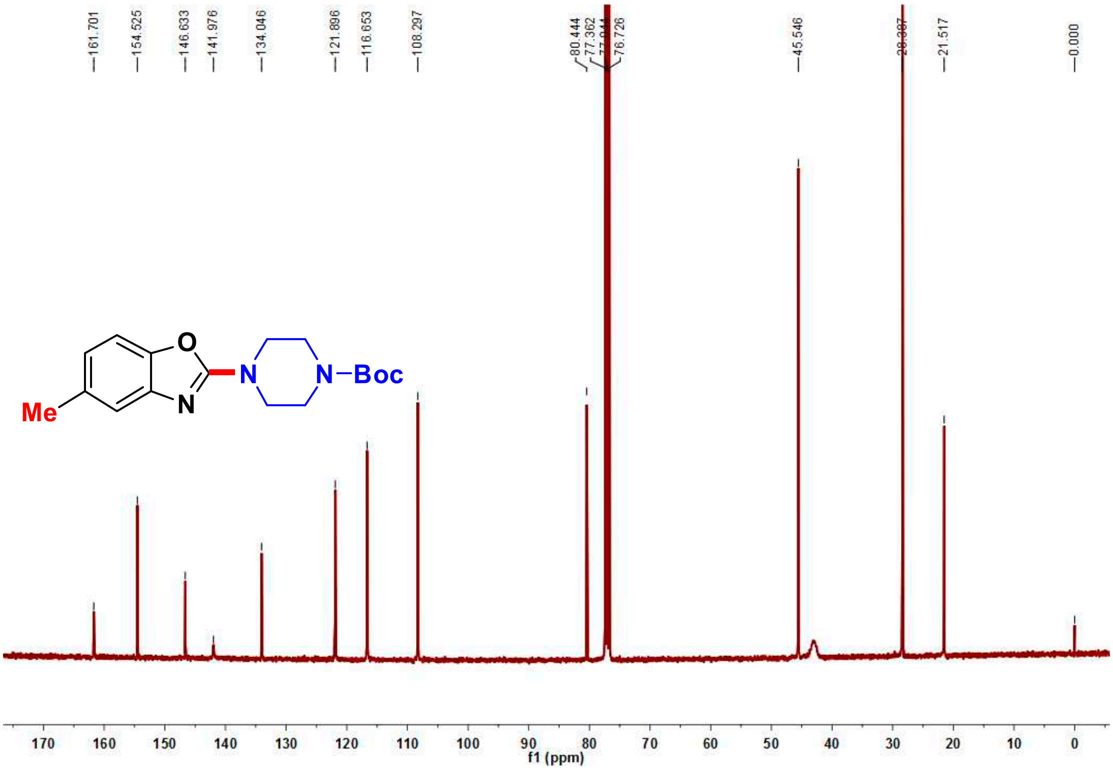

N,*N*-Diallyl-6-methylbenzo[*d*]oxazol-2-amine (4h)

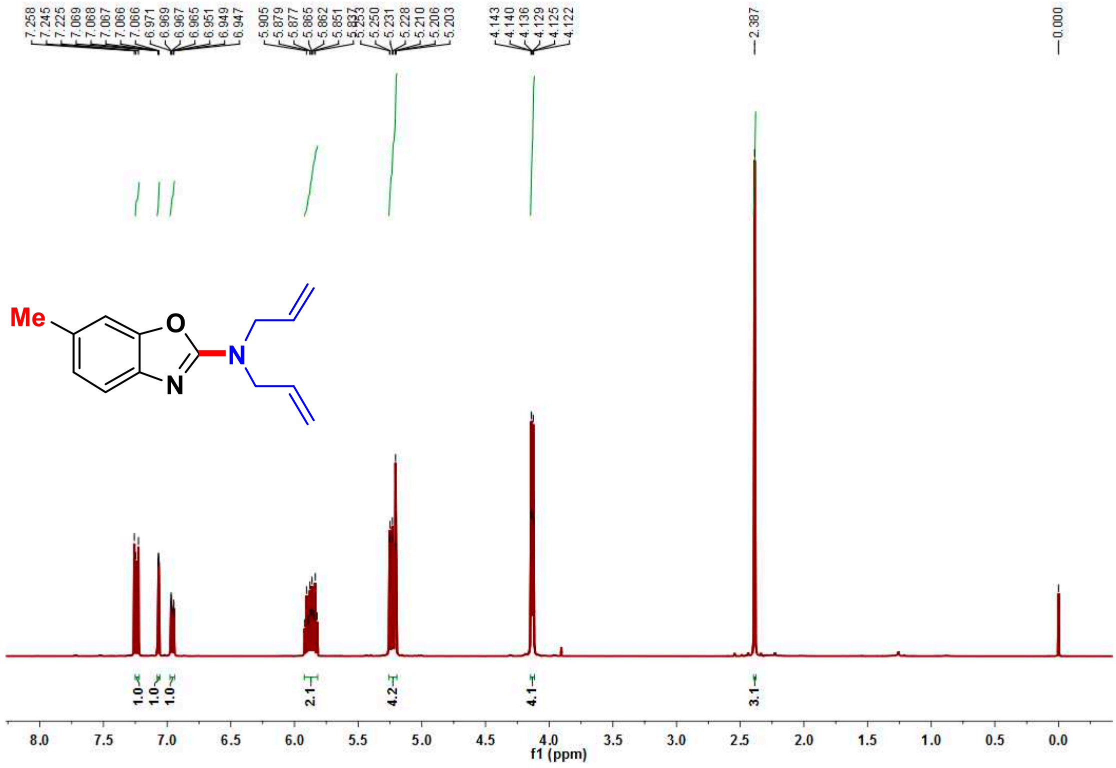

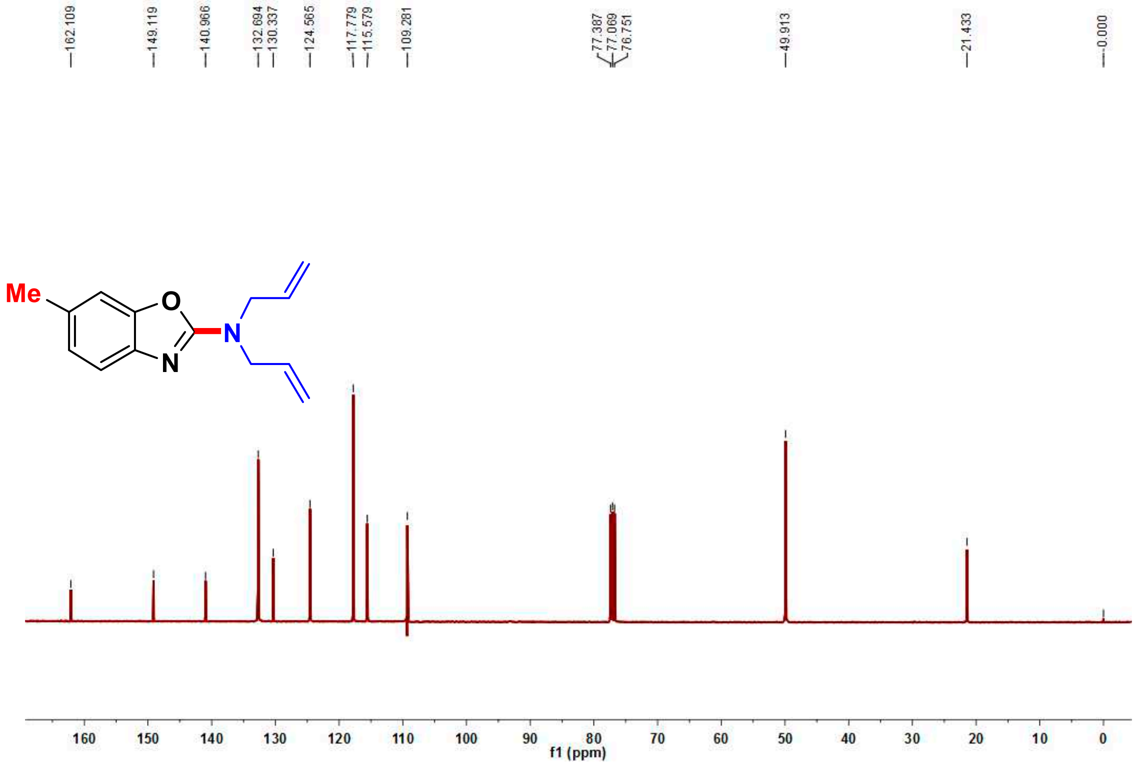

N,*N*-Dibenzyl-6-methylbenzo[*d*]oxazol-2-amine (4i)

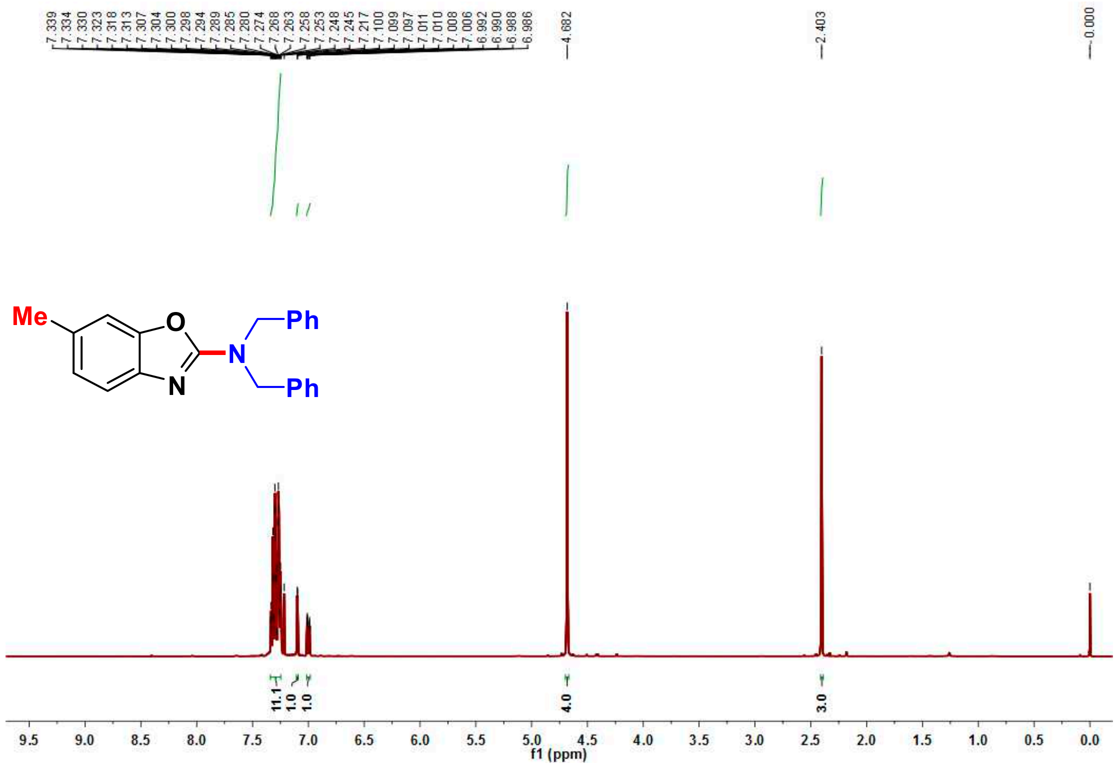

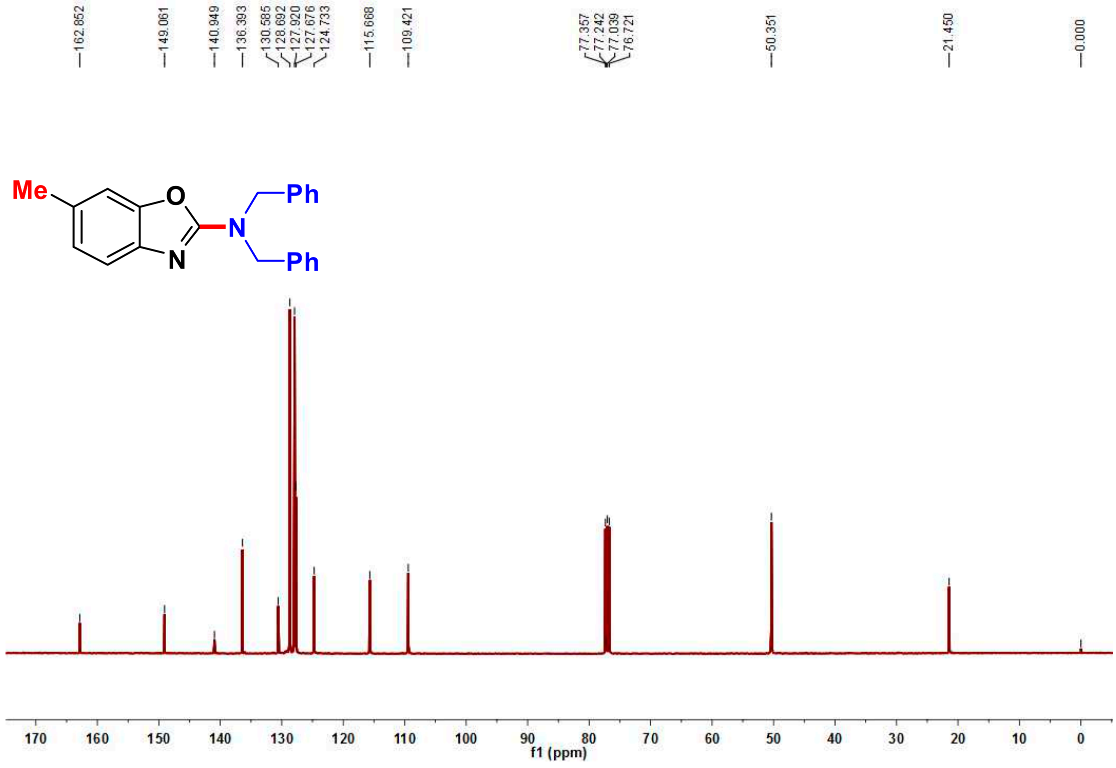

***Tert*-butyl 4-(6-methylbenzo[*d*]oxazol-2-yl)piperazine-1-carboxylate (4j)**

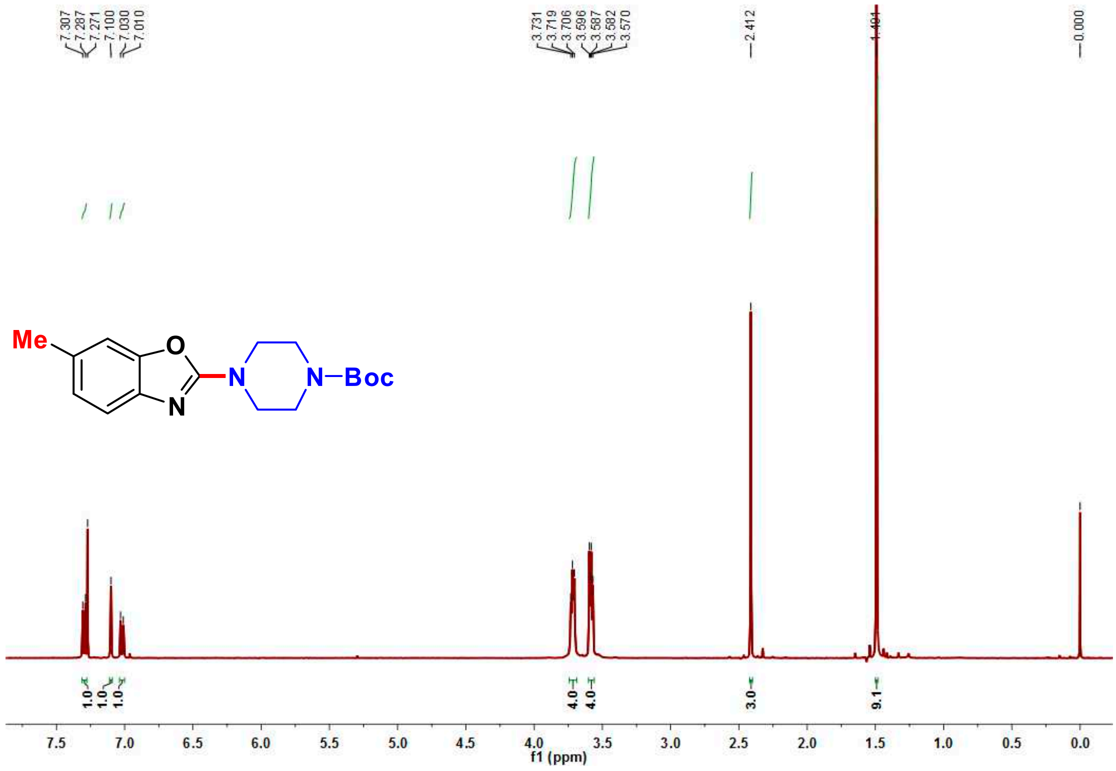

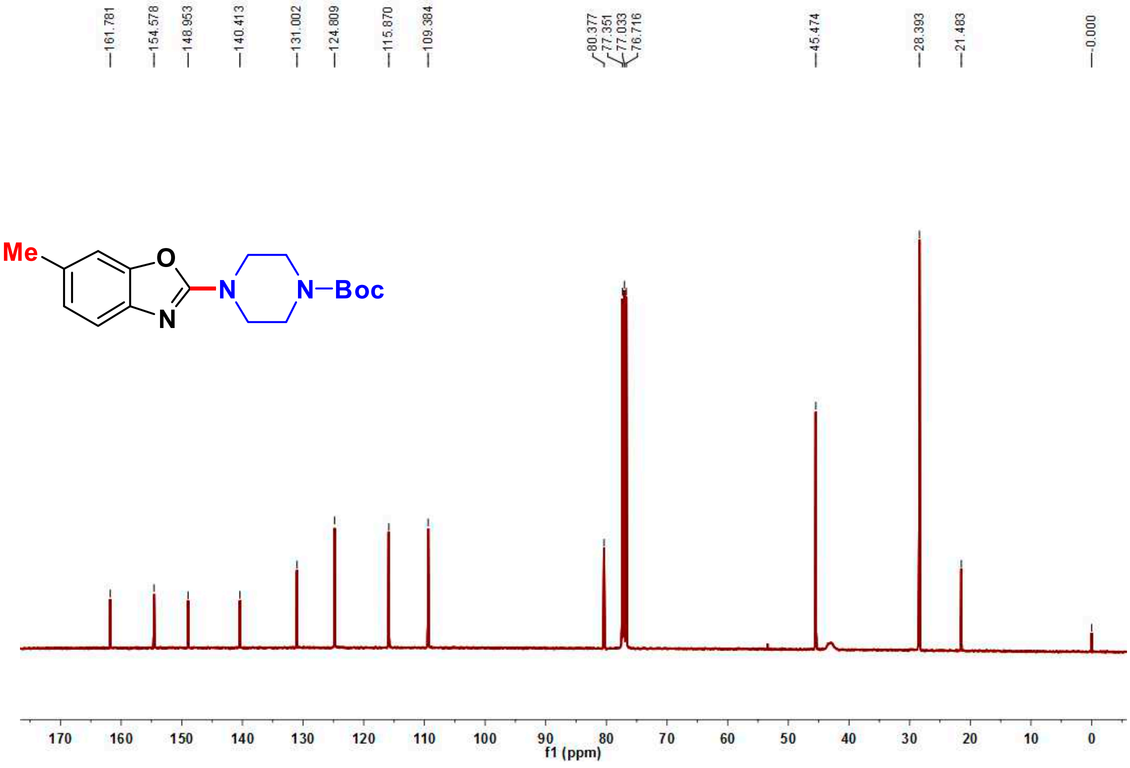

N,*N*-Diallyl-5-chlorobenzo[*d*]oxazol-2-amine (4k)

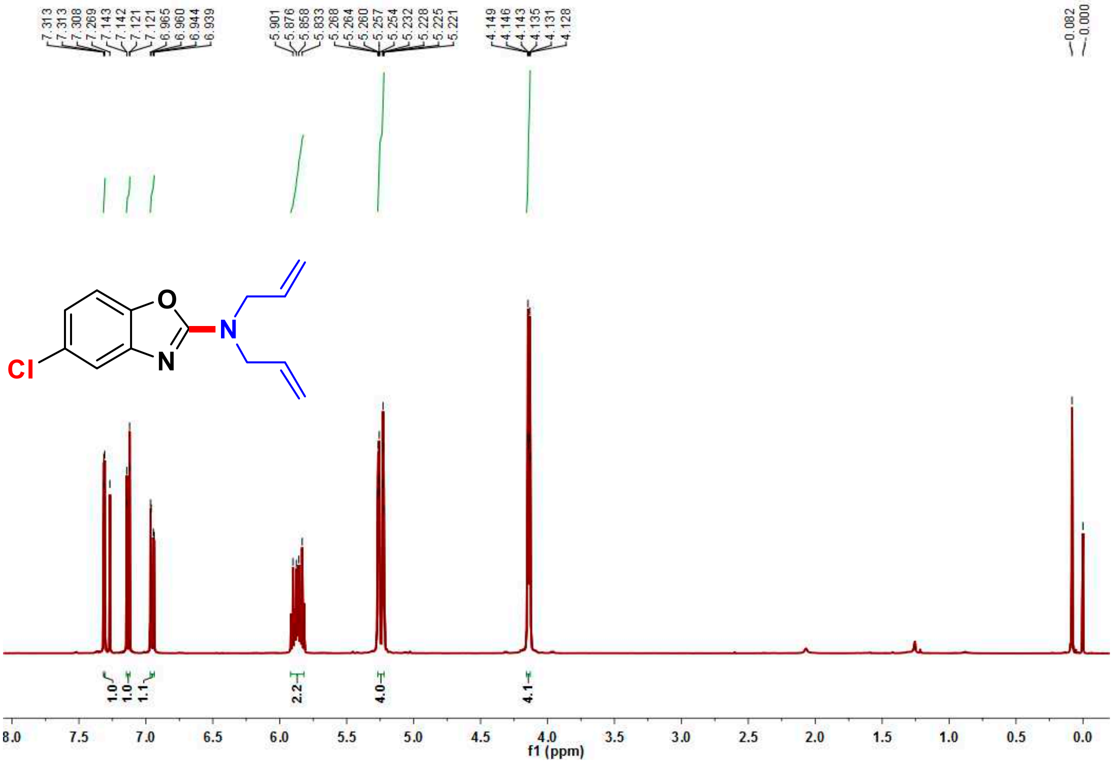

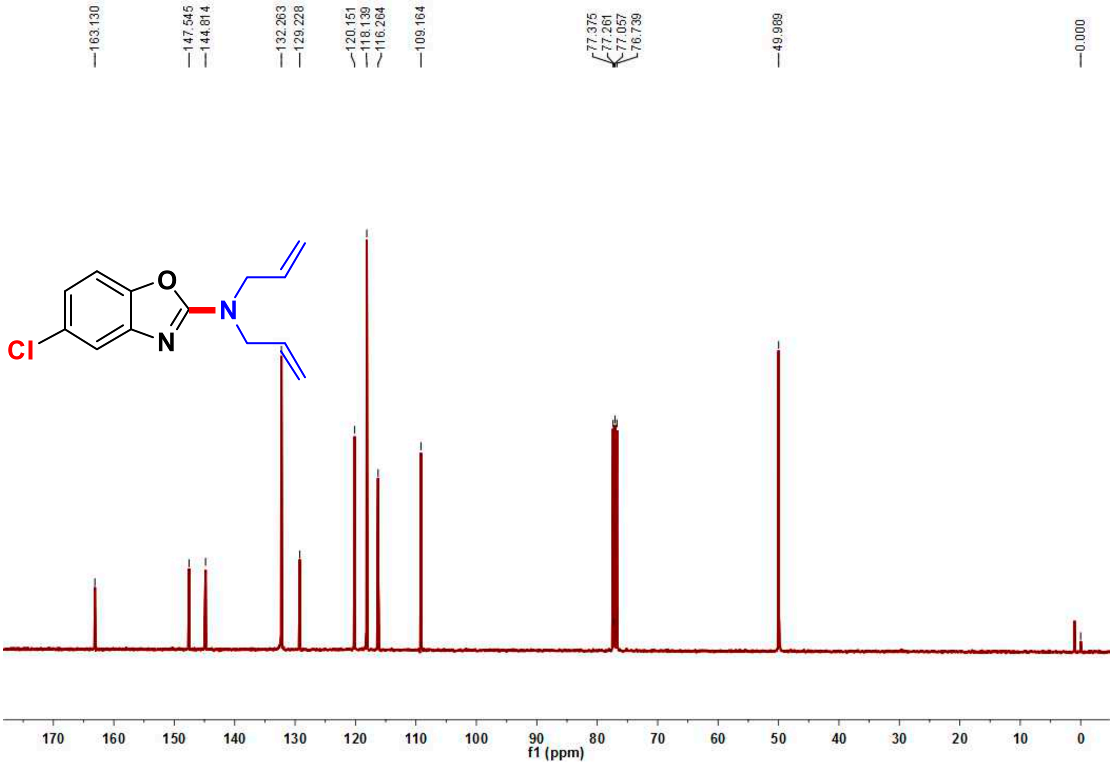

N,*N*-Dibenzyl-5-chlorobenzo[*d*]oxazol-2-amine (4l)

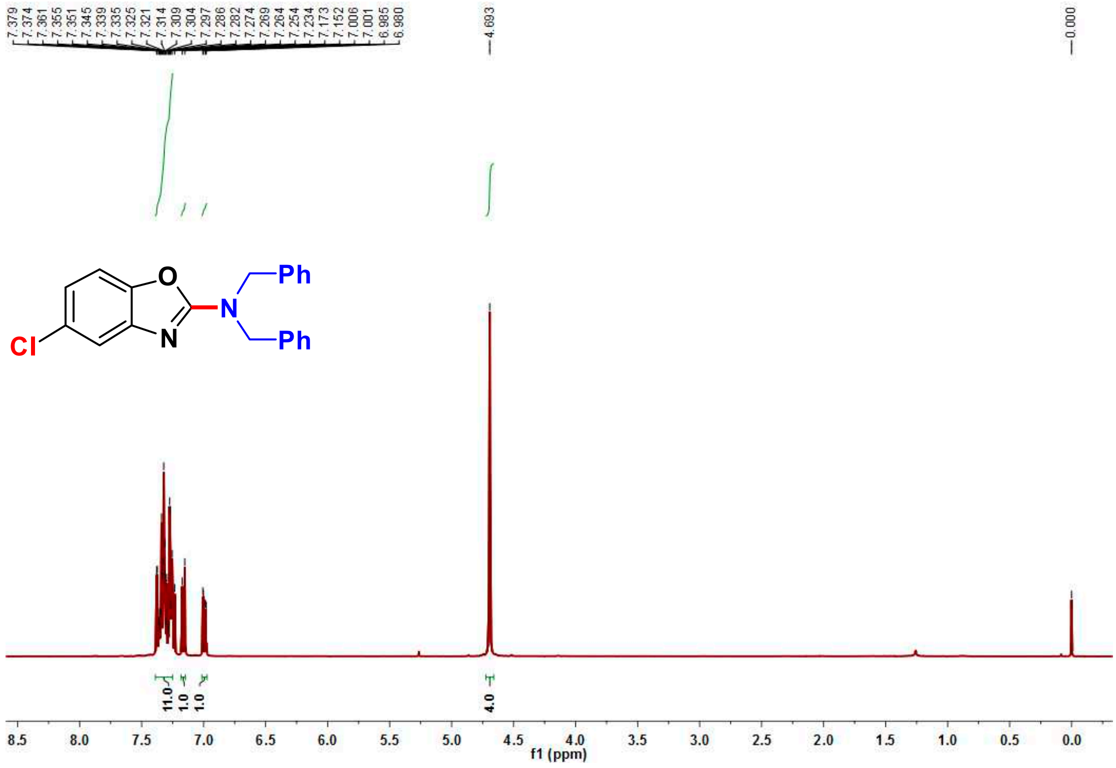

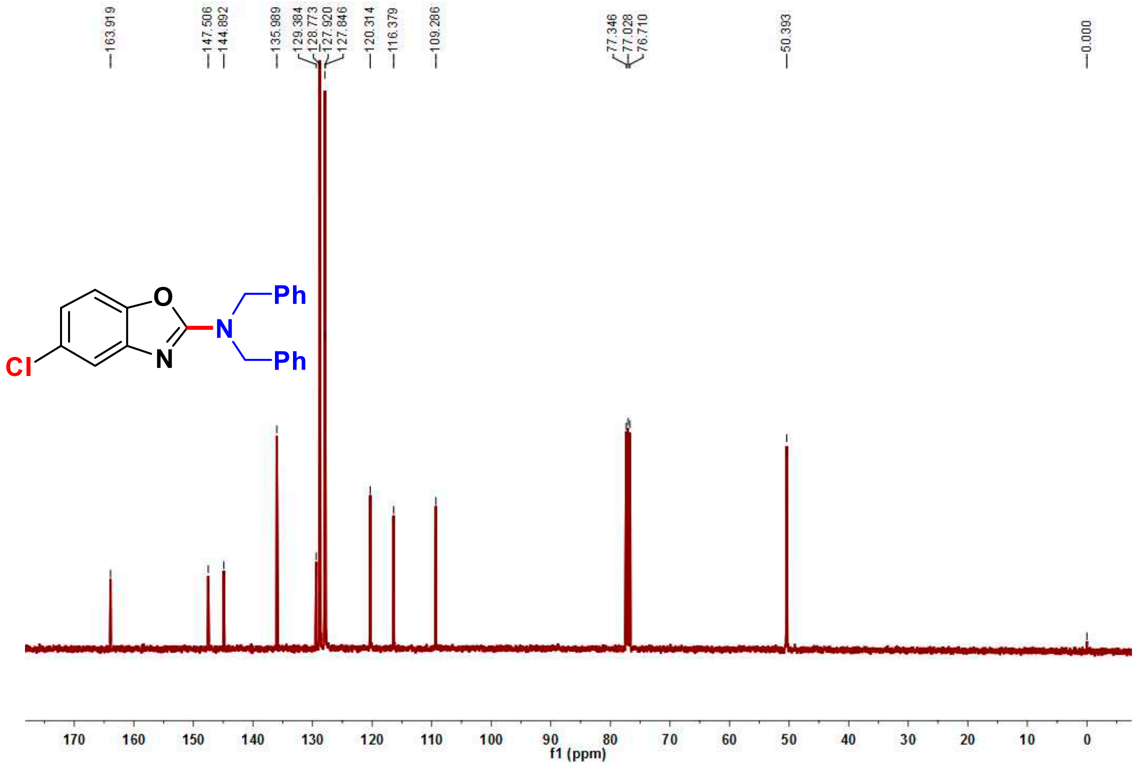

***Tert*-butyl 4-(5-chlorobenzo[*d*]oxazol-2-yl)piperazine-1-carboxylate (4m)**

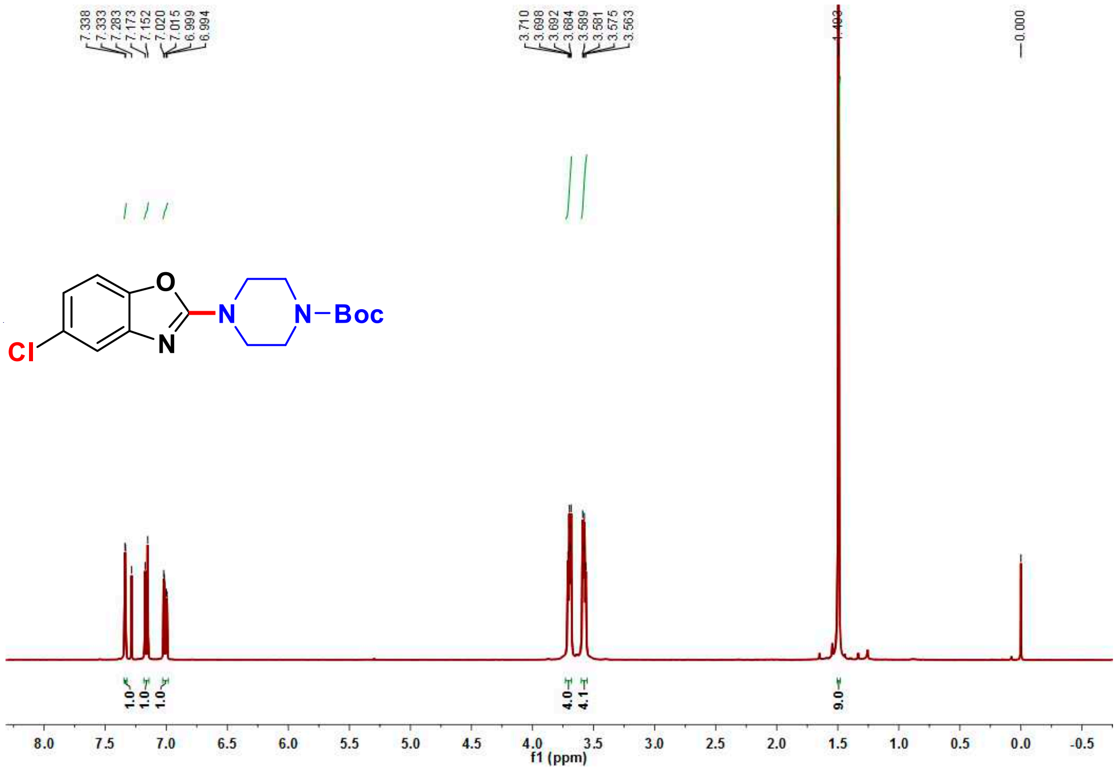

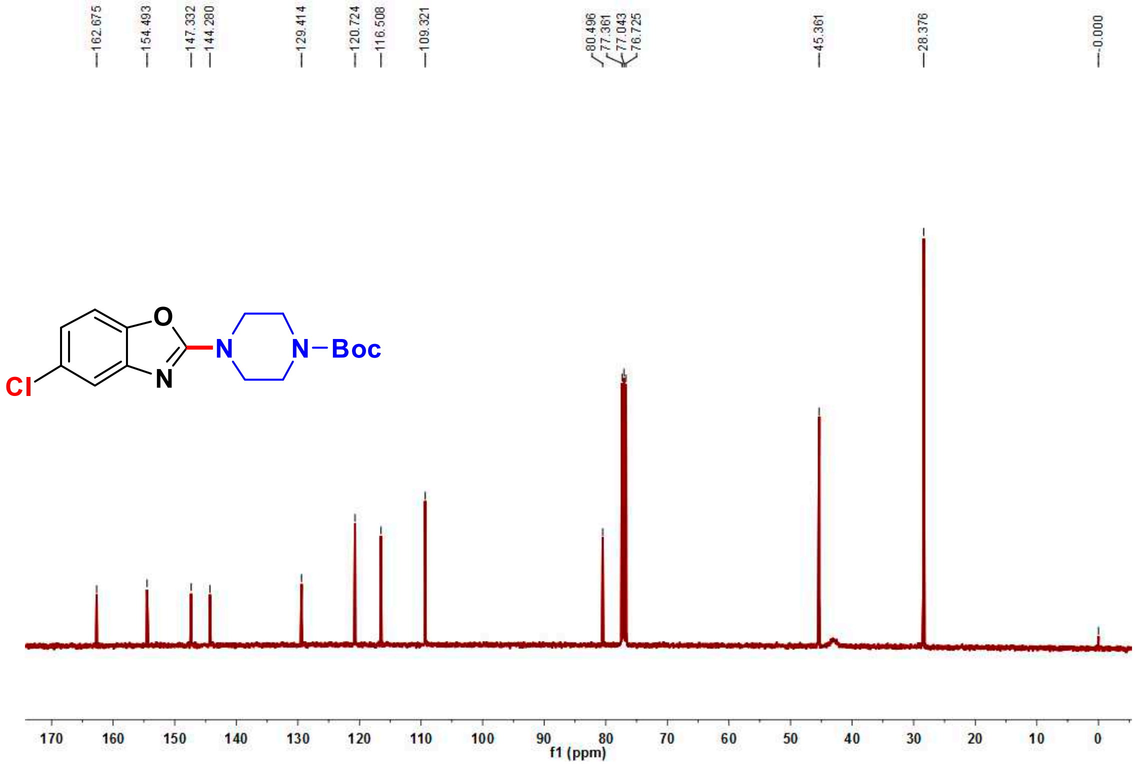

Supplement: Supplementary File 1 [file molecules-22-00576-s001.pdf]
